# Heat thermotherapy to improve cardiovascular function and cardiometabolic health: A systematic review and meta‐analysis

**DOI:** 10.1113/EP092404

**Published:** 2025-10-30

**Authors:** Ben S. Price, Samuel J. E. Lucas, Ashley P. Akerman, Rachel E. Gilworth, Rebekah A. I. Lucas

**Affiliations:** ^1^ School of Sport, Exercise and Rehabilitation Sciences University of Birmingham Birmingham UK; ^2^ Institute of Sport Manchester Metropolitan University Manchester UK; ^3^ Centre for Human Brain Health University of Birmingham Birmingham UK; ^4^ Ultromics Ltd Oxford UK

**Keywords:** blood pressure, cardiovascular disease, endothelial function, heat thermotherapy

## Abstract

Heat thermotherapy (HT) is reported to promote cardiovascular (CV) and cardiometabolic health benefits. This systematic review and meta‐analysis (CRD42020193669) empirically investigated the efficacy of HT in improving CV and cardiometabolic parameters by assessing responses to single versus multiple HT bouts. Databases (EMBASE, MEDLINE, and Web of Science) were searched up to January 2025 for HT studies investigating CV and cardiometabolic parameters. Inclusion criteria were adults aged ≥18 years, a passive heating stimulus with no exercise involved, and a control group comparison. Fifty‐one papers were included in the meta‐analysis, and publications were separated into HT single‐bout (1 heating bout) and HT multiple bouts (>1 heating bout). After removing outliers, HT reduced diastolic blood pressure in single (*n *= 20, −2 mmHg [−4, 0], *I* ^2^ = 76%) and multiple bouts (*n *= 9, −3 mmHg [−6, −1], *I* ^2^ = 56%) in comparison to control conditions. Mean arterial pressure was reduced in single (*n *= 22, −5 mmHg [−8, −3], *I* ^2^ = 63%) and multiple bouts (*n *= 6, −4 mmHg [−6, −2], *I* ^2^ = 49%). Systolic blood pressure was reduced in multiple bouts (*n *= 8, −5 mmHg [−9, −1], *I* ^2^ = 73%), whereas only single bouts improved flow‐mediated dilation (*n *= 11, 0.31 g [0.06, 0.56], *I* ^2^ = 0%), and total peripheral artery shear rate (*n *= 11, 4.09 g [2.87, 5.30], *I* ^2^ = 71%; all *P *< 0.05). C‐reactive protein, heat shock proteins and arterial stiffness did not change after single or multiple bouts (all *P >* 0.053). This meta‐analysis found HT improved some acute and chronic CV parameters, with the magnitude of improvement largely unaffected by an individual's health status or HT intervention duration.

## Abstract (highlights)

- **What is the central question of this study?**
  Does heat therapy (HT) improve cardiovascular and cardiometabolic parameters, and how do the effects differ between a single session and multiple sessions of HT?
- **What advances does it highlight?**
  Heat thermotherapy acutely reduced diastolic and mean arterial blood pressure, and increased peripheral artery shear rate and endothelial function. Multiple‐bouts resulted in longer‐term adaptations, including lower systolic, diastolic and mean arterial blood pressure and appear largely unaffected by health status or age. The current meta‐analysis supports the use of heat thermotherapy as a promising intervention for improving cardiovascular parameters such as blood pressure.

## 1 INTRODUCTION

Heat thermotherapy (HT) is the application of a passive (non‐exercising) heating stimulus that increases core body temperature (*T*_c_) and results in beneficial health outcomes (Brunt, Howard et al., 2016; Ely, Clayton et al., 2019; Naumann et al., 2020). Specifically, HT has been shown to reduce blood pressure (BP), inflammatory markers, fasting glucose, glycated haemoglobin, and improve endothelial function, all associated with a reduction in cardiovascular disease (CVD) and related mortality (Fiuza‐Luces et al., 2018; Ras et al., 2013). These HT‐related cardiovascular (CV) and cardiometabolic improvements have been observed in different populations, from young, sedentary and healthy cohorts (Brunt, Howard et al., 2016) to chronic heart failure patients (Kihara et al., 2002). It is unclear whether the magnitude of HT‐related improvement differs between populations.

Numerous narrative reviews have discussed how HT improves CV and metabolic health (Brunt & Minson, 2021; Cheng & MacDonald, 2019; Ely et al., 2018; Hoekstra et al., 2020). These reviews indicate the interest in, and the potential use of, HT as a therapeutic tool. However, these narrative reviews did not systematically review the literature or provide empirical evidence to support purported HT mechanisms. A previous meta‐analysis by Pizzey et al. (2021) examined resting BP and flow‐mediated dilation (FMD) responses to HT (specifically, >10 HT sessions) in healthy and clinical populations. A total of 12 papers were included in this meta‐analysis, which showed that repeated use of HT reduced BP and improved FMD. However, this meta‐analysis did not include studies examining acute BP and FMD responses. Subsequently, it remains unclear how acute HT responses translate to long‐term adaptations. Identifying acute responses to HT is essential in guiding future research and clinical application (i.e. optimising HT to enhance health).

Two previous systematic reviews have examined glycaemic HT responses (Maley et al., 2019; Sebok et al., 2021). Maley et al. (2019) found that glycaemic control was not affected in non‐diabetics but was acutely impaired in diabetics following HT. Meanwhile, Sebok et al. (2021) found that fasting glucose was unaffected in diabetic participants following HT. Inflammation has a strong association with glycaemic control and overall cardiometabolic health (Ely et al., 2018); thus, it would be advantageous to understand how it is affected by HT. However, key cardiometabolic responses (e.g., heat shock proteins; HSPs) and inflammatory markers such as C‐reactive protein (CRP) have not been systematically examined. Therefore, this systematic review and meta‐analysis empirically investigated the efficacy of HT in improving CV and cardiometabolic parameters by assessing responses to single versus multiple HT bouts.

## 2 METHODS

### 2.1 Ethical approval

This systematic review and meta‐analysis extracted only previously published data and did not involve any new data collection from human participants or animals.

### 2.2 Overview

This review followed the Preferred Reporting System for Systematic Reviews and Meta‐Analyses (PRISMA) guidelines (Moher et al., 2009). The protocol for this review is published on the PROSPERO register (https://www.crd.york.ac.uk/prospero) under the registration number CRD42020193669. Details of the search terms used for the current systematic review, alongside an example search for the Web of Science database, can be found in the Supporting information.

### 2.3 Information sources and search strategy

Bibliographic databases, MEDLINE, EMBASE and Web of Science, were primarily searched for relevant publications. A manual search (via Google Scholar) was then conducted to retrieve all relevant publications. Databases and manual searches included publications from the earliest start date to 5 January 2025. Search results were extracted to EndNote (Clarivate Analytics, Philadelphia, PA, USA), and duplicates were removed before continuing the screening process.

### 2.4 Study inclusion/exclusion process

The inclusion and exclusion process used the patient, intervention, comparison, and outcome (PICO) framework (Schardt et al., 2007). This PICO framework included studies that: examined adult human populations (≥18 years) with or without diagnosed health conditions; used a passive heat stress intervention (i.e. non‐exercising heat stimuli, such as hot water immersion (HWI) or sauna bathing) to increase limb or *T*_c_; used a randomised study design; and measured CV or cardiometabolic responses. Publications were excluded if the heat stimulus occurred in addition to exercise, pharmaceutical interventions, any other concurrent intervention, or if there was no control comparison. The CV parameters included for this meta‐analysis were blood pressure, arterial stiffness, FMD, and shear rate. The cardiometabolic parameters were interleukin‐6 (IL‐6), glucose (including fasted, mixed meal and oral glucose tolerance test (OGTT), grouped due to the limited number of reported values), HSPs (grouped due to the limited number of reported values), and CRP. Cardiometabolic data for IL‐6 were derived from either serum or plasma, while HSP data came from muscle, plasma, serum and adipose tissues. To account for heterogeneity within the cardiometabolic protocols (i.e. serum vs plasma), the data were presented as a standardised mean difference in the meta‐analysis.

Titles and abstracts of citations identified in the search were independently screened by reviewers (B.P. and R.G.), according to the inclusion/exclusion criteria above. To ensure the publication selection procedure was applied consistently, a random sample of 20% of the citations was screened by both reviewers (B.P. and R.G.), and the results were compared. Any disagreements regarding a study's eligibility were discussed with a third reviewer (R.L.).

### 2.5 Data extraction

Data from included full texts were extracted by two reviewers (B.P. and R.G.), including data on participant demographics, study design, intervention design, *T*_c_ changes, and cardiovascular and cardiometabolic outcomes. To minimise unit‐of‐analysis errors from multiple glucose measures, changes in fasting glucose (pre‐to‐post) were prioritised. If unavailable, postprandial or OGTT‐derived changes were used. Fasting glucose values were extracted from the time point nearest to the intervention's end; for OGTT or mixed‐meal tests, the peak postprandial glucose value was extracted. Extracted data were checked by an additional reviewer (A.A.) before analysis. The mean difference and standard deviation/standard error of the mean were extracted from all eligible studies. Where possible, results were expressed as absolute values (mean ± SD), with authors contacted via email to retrieve any missing data. If publications did not report results as a mean difference with 95% confidence intervals, the *Cochrane Handbook* method (7.7.7.2) for calculating the standard difference from 95% confidence intervals was applied (Li et al., 2019). In studies reporting non‐parametric results (i.e. median and interquartile ranges), the mean and standard deviation were estimated from the sample size, median and interquartile range (Wan et al., 2014). If unavoidable, data presented in figures alone were extracted using an online software package (WEBPLOT DIGITIZER; https://apps.automeris.io/wpd/).

### 2.6 Risk of bias

A risk of bias assessment (Cochrane RoB 2; Higgins, 2011) was conducted using COCHRANE Guidelines (Sterne et al., 2019). This assessment determined the risk of bias arising from the (1) randomisation process, (2) deviations from intended interventions, (3) missing outcome data, (4) measurement of the outcome, and (5) selection in the reported result. One reviewer completed the risk of bias (B.P.), with a second reviewer (R.L.) consulted as required.

### 2.7 Data synthesis and analysis

The current study aimed to distinguish between acute (e.g. min/h after a HT bout) and chronic (e.g. days following a HT intervention) CV and cardiometabolic responses to HT. Therefore, extracted data were divided into two discrete categories (i.e. single‐bout (1 heating bout) and multiple bouts (>1 heating bout)) to reduce the possibility of a unit of analysis error and appropriately represent the physiological process within the data.

For single‐bout and multiple‐bout categories, mean differences between intervention and control groups were calculated, and overall effect estimates (raw effect or Hedges’ *g*) were calculated using generic inverse variance models and random effect models. Hedges’ *g* values of 0.15, 0.40 and 0.70 indicate small, medium and large effect sizes, respectively (Lovakov & Agadullina, 2021). Estimated significance (*P*‐value; α > 0.05) and heterogeneity (*I* ^2^) were examined, with *I* ^2^ > 50% and *I* ^2^ > 75% indicative of substantial and considerable heterogeneity, respectively (Higgins et al., 2003). Where heterogeneity was substantial (*I* ^2^ ≥ 50%), subgroup analysis was performed to investigate variables (i.e. heating modality, duration of HT bout, and participant demographics). Additionally, a meta‐regression was completed for each CV or cardiometabolic parameter when applicable.

The meta‐regression investigated whether greater cumulative exposure to HT was associated with a greater magnitude of effect for CV and cardiometabolic parameters. To quantify the exposure to HT, the cumulative minutes were calculated as the product of the duration of the HT session and the total number of sessions completed. Data on session duration and frequency were extracted from included publications, and cumulative minutes were used as the independent variable in the meta‐regression analysis.

The Egger test, trim and fill method, and *p*‐curve analysis assessed small study effects (including potential publication bias). The impact of influential points on the pooled summary effect size was estimated with an influence analysis using multiple indicators (DFFITS, Cook's distance, and covariance ratio). To avoid unit‐of‐analysis errors, control groups from multi‐arm trials were appropriately split (Deeks et al., 2019). All statistical analyses were completed using packages (Tidyverse (Wickham et al., 2019), Meta (Balduzzi, 2019), Metafor (Viechtbauer, 2010) and Dmetar (Harrer et al., 2019) written for R (Core Team, 2014) and implemented in RStudio (Allaire, 2012).

## 3 RESULTS

### 3.1 Systematic search

**FIGURE 1:**
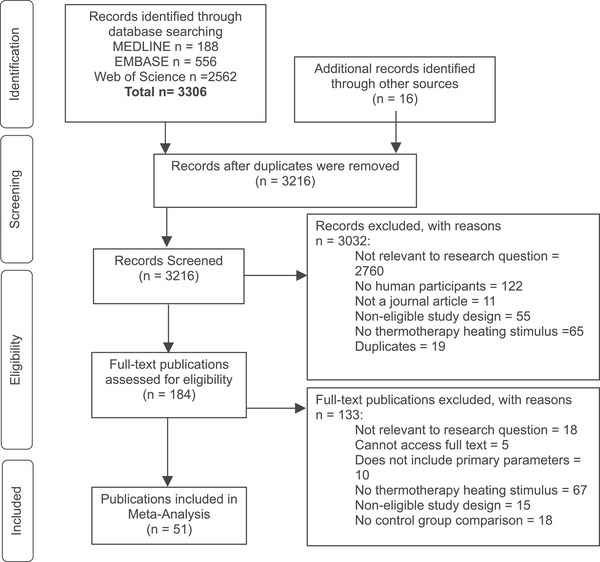
PRISMA flow diagram showing the search, screening and selection process for included publications in the meta‐analysis.

Figure 1 shows the search, screening and selection process for eligible publications. In total, 51 peer‐reviewed publications were included, and their data were extracted and included in the meta‐analysis as shown in Tables 1 and 2.

**TABLE 1:** Summary of heat thermotherapy intervention characteristics, measurement parameters and participant demographics from all included studies (*k* = 51).

Intervention characteristics							Parameters		Participant demographics		
Author	Heating modality	Body surface heated	Temperature (°C)	Bout duration (min)	Number of bouts	Cumulative minutes	Cardiovascular	Cardiometabolic	Age	CVD Risk Factors	Control
Akerman et al. (2019)	HWI	≥ Shoulder	39	30	60	1800	DBP, SBP & MAP, FMD	n/a	Older	Y	N
Amin et al. (2021)	HWI	≥ Mid‐sternum	42	30	1	30	DBP, SBP, MAP SR	n/a	Young	N	N
Bailey et al. (2016)	HWI	≥ Top‐sternum	42	30	24	720	DBP, SBP & MAP, FMD	n/a	Young	N	N
Behzadi et al. (2020)	Sauna	n/a	80 (20% RH)	20	1	20	n/a	IL‐6	Older	N	Y
Behzadi et al. (2022)	HWI	≥ Neck then waist	41	90	1	90	DBP, SBP FMD	HSP glucose	Older	Y	N
Brunt, Howard et al. (2016)	HWI	≥ Shoulder then waist	40.5	90	36	3240	DBP, SBP & MAP, FMD	n/a	Young	N	Y
Brunt, Jeckell et al. (2016)	HWI	Shoulder then waist	40.5	60	1	60	FMD	n/a	Young	N	Y
Campbell et al. (2022)	HWI/sauna	≥ Nipple then neck	40 HWI 55 (54% RH) Sauna	60	1	60	DBP, SBP & MAP	n/a	Young	N	Y
Cheng et al. (2019)	Heating Pad	Forearm	42	10	1	10	DBP, SBP & MAP, FMD	n/a	Young	N	Y
Cheng et al. (2021)	HWI	Ankle/knee	45	45	1	45	DBP, SBP & MAP, SR & FMD	IL‐6 HSP	Young	N	Y
Cheng et al. (2024)	HWI	Ankle	42.8	45	24	1400	DBP, SBP & MAP FMD PWV	n/a	Young	N	Y
Coombs et al. (2021)	Water‐perfused suit	Whole‐ body/forearm	49	60	1	60	MAP SR & FMD	n/a	Young	N	Y
Debray et al. (2023)	Sauna	n/a	79 (13% RH)	30	32	960	DBP & SBP FMD	n/a	Older	Y	Y
Ely et al. (2019 a,b)	HWI	≥ Shoulder then waist	40.5	60	30	1800	DBP, SBP & MAP FMD PWV	IL‐6 Glucose	Young	Y	Y
Engelland et al. (2019)	HWI	Both legs	40.5	60	1	60	MAP SR & FMD	n/a	Young	N	Y
Fatahi et al. (2023)	Sauna	n/a	Not reported	60	36	2160	n/a	Glucose	Middle Aged	N	Y
Faulkner et al. (2017)	HWI	≥ Waist	40.2	60	1	60	n/a	IL‐6 HSP	Young	Y	N
Francisco et al. (2021)	HWI	≥ Clavicle then sternum	40.5	60	1	60	DBP, SBP & MAP SR	n/a	Young	N	N
Freemas et al. (2024)	Water‐perfused suit	Whole‐body	50	270	1	270	MAP	n/a	Young	N	Y
Gayda et al. (2012)	Sauna	n/a	85 (50% RH)	16	1	16	DBP & SBP	n/a	Older	Y	Y
Gravel et al. (2019)	Sauna	n/a	80.2 (23% RH)	20	1	20	DBP, SBP & MAP FMD	n/a	Older	N	Y
Hedley et al. (2002)	Sauna	n/a	65 (15% RH)	30	1	30	DBP, SBP & MAP	n/a	Young	N	Y
Hemingway et al. (2022 a,b)	Water‐perfused suit	Whole‐body	48	71	1	71	MAP SR & FMD	HSP	Young & Older	N (Young) Y (Older)	Y
Hoekstra et al. (2018)	HWI	Neck	60	39	10	600	DBP & SBP	IL‐6 Glucose HSP	Young	Y	Y
Hoekstra et al. (2021)	Water‐perfused suit	Whole‐body/legs	50	90	1	90	DBP & SBP SR	IL‐6 Glucose	Young	N	Y
Hu et al. (2012)	HWI	Feet	43	30	1	30	DBP, SBP	n/a	Young & Older	N	Y
Iguchi et al. (2012)	Sauna	n/a	73 (< 10% RH)	30	1	30	DBP & SBP	HSP	Young	N	Y
Imamura et al. (2001)	Sauna	n/a	60 (RH not reported)	15	14	210	SBP FMD	Glucose	Middle Aged	Y	Y
James et al. (2021)	HWI	≥ Clavicle then waist	40	60	1	60	DBP & SBP	Glucose HSP	Older	Y	Y
Kihara et al. (2002)	Sauna	n/a	60 (RH not reported)	15	14	210	DBP & SBP FMD	n/a	Older	Y	Y
Kimball et al. (2018)	Sauna	n/a	73 (10% RH)	30	1	30	n/a	Glucose	Young	N	Y
Kojima et al. (2018)	HWI	≥ Neck	42	20	1	20	MAP	n/a	Young	N	Y
Leicht et al. (2019)	HWI	≥ Sterno‐clavicular notch	39.2	60	1	60	n/a	Glucose	Young	N	Y
Maley et al. (2023)	HWI	≥ Clavicle then waist	40.3	120	1	120	DBP & SBP	Glucose	Young	N	Y
Masuda et al. (2004)	Sauna	n/a	60 (RH not reported)	15	14	210	DBP & SBP	Glucose	Middle Aged	Y	Y
McGarity‐Shipley et al. (2021)	HWI	Single leg	42.5	35	40	1400	DBP, SBP & MAP FMD	n/a	Young	N	Y
Monroe et al. (2020)	Water‐perfused suit	Both legs	43	90	1	90	DBP, SBP & MAP	n/a	Older	Y	Y
Monroe et al. (2021)	Water‐perfused suit	Both legs	43	90	1	90	DBP & SBP	n/a	Older	Y	Y
Neff et al. (2016)	HWI	Both legs	48	90	1	90	DBP, SBP & MAP	IL‐6	Older	Y	Y
Ogawa et al. (2021)	Water‐perfused suit	Whole‐body/legs	50	90	1	90	DBP & SBP SR	n/a	Young	N	Y
Olah et al. (2011)	HWI	Not reported	38	30	15	450	n/a	CRP Glucose	Older	Y	Y
Oyama et al. (2013)	HWI	≥ Xiphoid process	40	10	10	100	DBP, SBP & MAP	CRP IL‐6	Older	Y	Y
Qiu et al. (2014)	HWI	Whole‐body	39	20	28	560	n/a	Glucose	Older	Y	Y
Romero et al. (2017)	HWI	Ankle	42	45	1	45	MAP SR & FMD	n/a	Young & Older	N (young) Y (Older)	Y
Roxburgh et al. (2023)	HWI	≥ Mid‐sternum	40	30	36	1080	DBP, SBP & MAP	n/a	Older	Y	N
Sanchez et al. (2024)	HWI	Feet/calves	40	120	1	120	DBP, SBP & MAP	Glucose	Young	N	Y
Schenaarts et al. (2024)	Sauna	n/a	60 (RH not reported)	40	1	40	n/a	Glucose	Older	Y	Y
Steward et al. (2024)	HWI	≥ Mid‐sternum	40	70	1	70	DBP, SBP & MAP SR	IL‐6	Middle Aged	N	N
Teixeira et al. (2017)	HWI	Single foot	42	30	15	450	FMD	n/a	Young	N	Y

*Note*: Ely et al. (2019 a,b) and Hemingway et al. (2022 a,b) refer to papers published in the same year, each using largely distinct datasets derived from a single overarching experimental study. If more than 1 CVD risk factor, then Y was selected; if fewer than one risk factor, then N was chosen for the health status column. Young (< 35 years), middle‐aged (36–59 years) and older (> 60 years). Abbreviations: CRP, C‐reactive protein; CVD, cardiovascular disease; DBP, diastolic blood pressure; FMD, flow‐mediated dilation; HSP, heat shock protein; HWI, hot water immersion; IL‐6, interleukin 6; MAP, mean arterial pressure; RH, relative humidity; SBP, systolic blood pressure; SR, shear rate. k denotes number of records.

**TABLE 2:** Summary subgroup and meta‐regression data for cardiovascular and cardiometabolic responses to a heat thermotherapy intervention.

Parameter	Publications (*n*)*	Effect estimate*	P*	Publications outliers removed (*n*)	Effect estimate after outliers removed	*P*	Heating modality subgroup* (*P*)	Control condition subgroup* (*P*)	Health status subgroup* (*P*)	Meta‐regression * removed	Meta‐regression* (*P*)
**Single bout response**											
** DBP (mmHg)**	26	−5 [−8 to −2]	<0.01	6	−2 [−4 to −0]	0.042	<0.01	0.301	N/A	0.70	0.210
** SBP (mmHg)**	26	−3 [−9 to 3]	0.336	11	−3 [−7 to 0]	0.082	0.633	0.059	N/A	0.59	0.992
** MAP (mmHg)**	24	−7 [−10 to −4]	<0.01	2	−5 [−8 to −3]	<0.01	0.148	0.226	N/A	0.55	0.803
** FMD**	12	0.40 [0.06 to 0.74]	0.026	1	0.31 [0.06 to 0.56]	0.019	0.031	N/A	N/A	0.28	0.590
** Shear rate**	16	4.36 [2.04 to 6.67]	<0.01	5	4.09 [2.87 to 5.30]	<0.01	N/A	0.476	N/A	0.93	0.828
** Glucose**	11	0.35 [−0.20 to 0.90]	0.19	0	0.35 [−0.20 to 0.90]	0.19	0.043	N/A	N/A	−0.11	0.618
** HSP**	11	−0.14 [−0.47 to 0.20]	0.373	0	−0.14 [−0.47 to 0.20]	0.373	N/A	0.517	N/A	0.37	0.341
** IL‐6**	9	−0.12 [−0.66 to 0.42]	0.628	0	−0.12 [−0.66 to 0.42]	0.628	<0.01	0.032	N/A	0.51	0.284
**Multiple bouts response**											
** DBP (mmHg)**	9	−3 [−6 to −1]	0.011	0	−3 [−6 to −1]	0.011	N/A	0.822	0.900	0.70	0.526
** SBP (mmHg)**	9	−4 [−8 to 0]	0.037	1	−5 [−9 to −1]	0.018	N/A	0.388	0.569	0.75	0.958
** MAP (mmHg)**	6	−4 [−6 to −2]	<0.01	0	−4 [−6 to −2]	<0.01	N/A	0.584	<0.01	0.70	0.963
** FMD**	9	1.04 [‐0.43 to 2.52]	0.141	1	0.44 [−0.1 to 0.97]	0.095	N/A	0.111	0.357	0.71	0.784
** Glucose**	8	−0.28 [−1.17 to 0.61]	0.478	1	0.02 [−0.23 to 0.27]	0.849	N/A	N/A	N/A	0.47	0.064
** HSP**	5	0.29 [−0.49 to 1.07]	0.358	0	0.29 [−0.49 to 1.07]	0.358	N/A	N/A	N/A	0.50	0.281
** IL‐6**	2	−0.53 [−0.97 to −0.08]	0.042	0	−0.53 [−0.97 to −0.08]	0.042	N/A	N/A	N/A	N/A	N/A
** CRP**	4	−0.61 [−1.87 to 0.65]	0.219	0	−0.61 [−1.87 to 0.65]	0.219	N/A	N/A	N/A	0.70	0.331
**Combination of single and multiple bouts responses**											
** Arterial Stiffness**	11	−0.43 [−0.86 to 0.01]	0.053	0	−0.43 [−0.86 to 0.01]	0.053	N/A	0.211	N/A	0.70	0.794

*Note*: *Outliers included. Note: Multiple−bouts refer to publications examining responses to > 1 heat thermotherapy bouts; Combined refers to single‐bout and multiple‐bout studies (i.e. for arterial stiffness values). The glucose parameter includes both fasting and postprandial responses pre‐post HT. The heat shock protein parameter includes both intracellular and extracellular heat shock proteins. Blood pressure values are expressed as mmHg, whilst all other variables are expressed as an effect size (Hedges’ *g*). Abbreviations: CRP, C‐reactive protein; DBP, diastolic blood pressure; FMD, flow‐mediated dilation; HSP, heat shock protein; IL‐6, interleukin 6; MAP, mean arterial pressure; N/A, not applicable; SBP, systolic blood pressure.

### 3.2 Publication characteristics

Of the 51 included publications, 11 were conducted in Europe (UK, Hungary, Netherlands and Austria), eight in East Asia (Japan and China), 27 in the Americas (USA, Canada and Brazil), four in Australasia (Australia and New Zealand) and one in the Middle East (Iran). Across all publications included, there was a total of 1055 participants. Participants were classified as healthy (no CV or cardiometabolic risk factors; *n* = 535) or unhealthy (>1 CV or cardiometabolic health risk factor; *n* = 520). The following demographic information was also extracted: participant age (young [18–35 years, *n* = 427], middle‐aged [36–59 years, *n* = 87], or older [>60 years, *n* = 541]) and sex (male, *n* = 647; female, *n* = 408). Within the 51 included publications, some participants were reported to have taken medication (including oral contraceptives, angiotensin‐converting enzyme inhibitors, statins, antiplatelets, beta‐blockers, corticosteroids and antidepressants), and this information was also extracted. Included publications used four passive heating modalities: specifically, 31 HWI, 13 sauna, eight water perfusion suit and one heating pad intervention.

### 3.3 Heat thermotherapy effects on CV and cardiometabolic parameters

The meta‐analysis included 31 HT single‐bout and 21 HT multiple‐bouts studies. The median HT duration in single‐bout studies was 60 min (interquartile range: 41 min). For multiple‐bouts studies, the number of HT bouts ranged from 2 to 60, with a median HT cumulative duration of 660 min (1062 min). All pooled effect sizes and subgroup data are presented in Table 2 and summarised qualitatively below.

**FIGURE 2:**
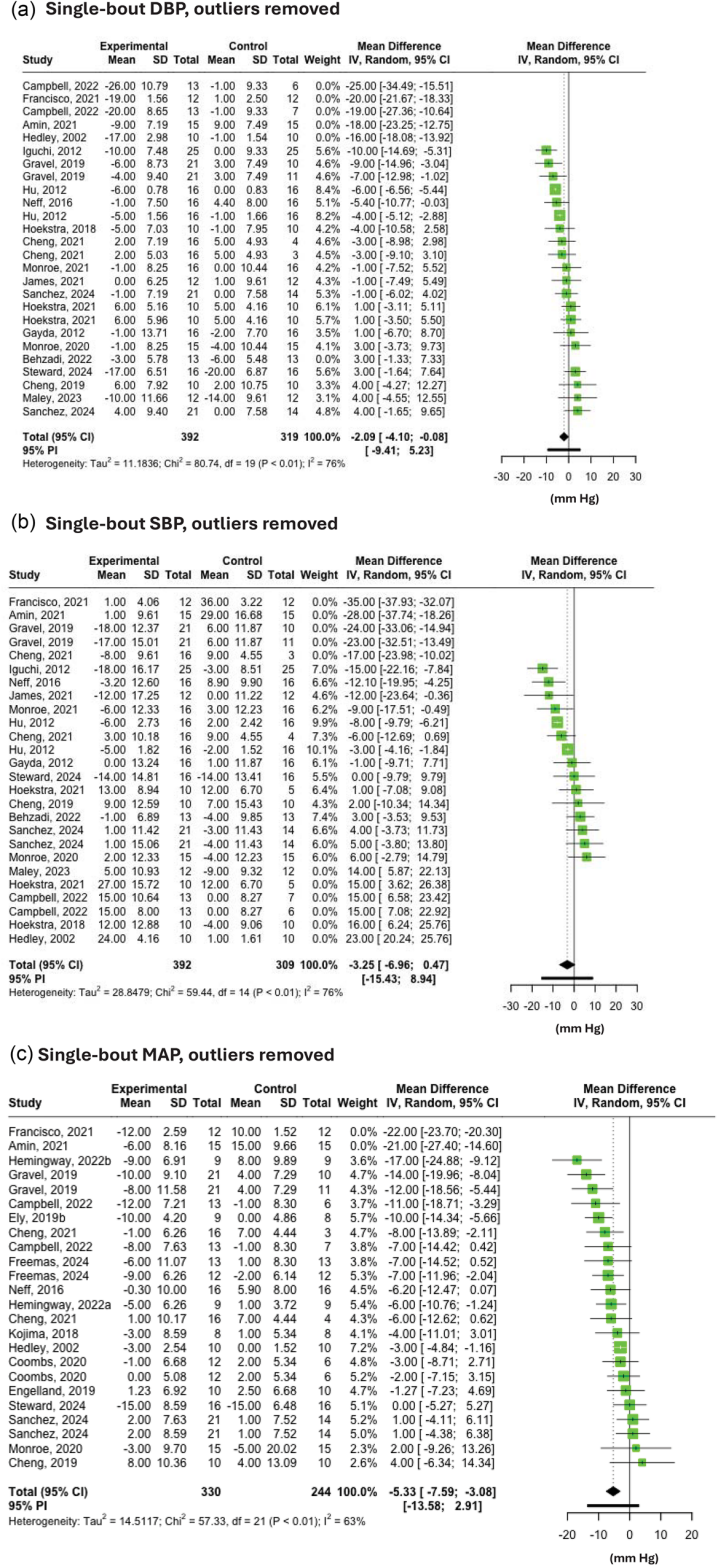
Pooled effect estimate of blood pressure response to a single bout of heat thermotherapy (HT) after removal of publication outliers. (a) Diastolic blood pressure (DBP) response; (b) systolic blood pressure (SBP); (c) mean arterial pressure (MAP) response. Mean differences in the delta change (pre–post) between the intervention and control arms are presented in mmHg.

**FIGURE 3:**
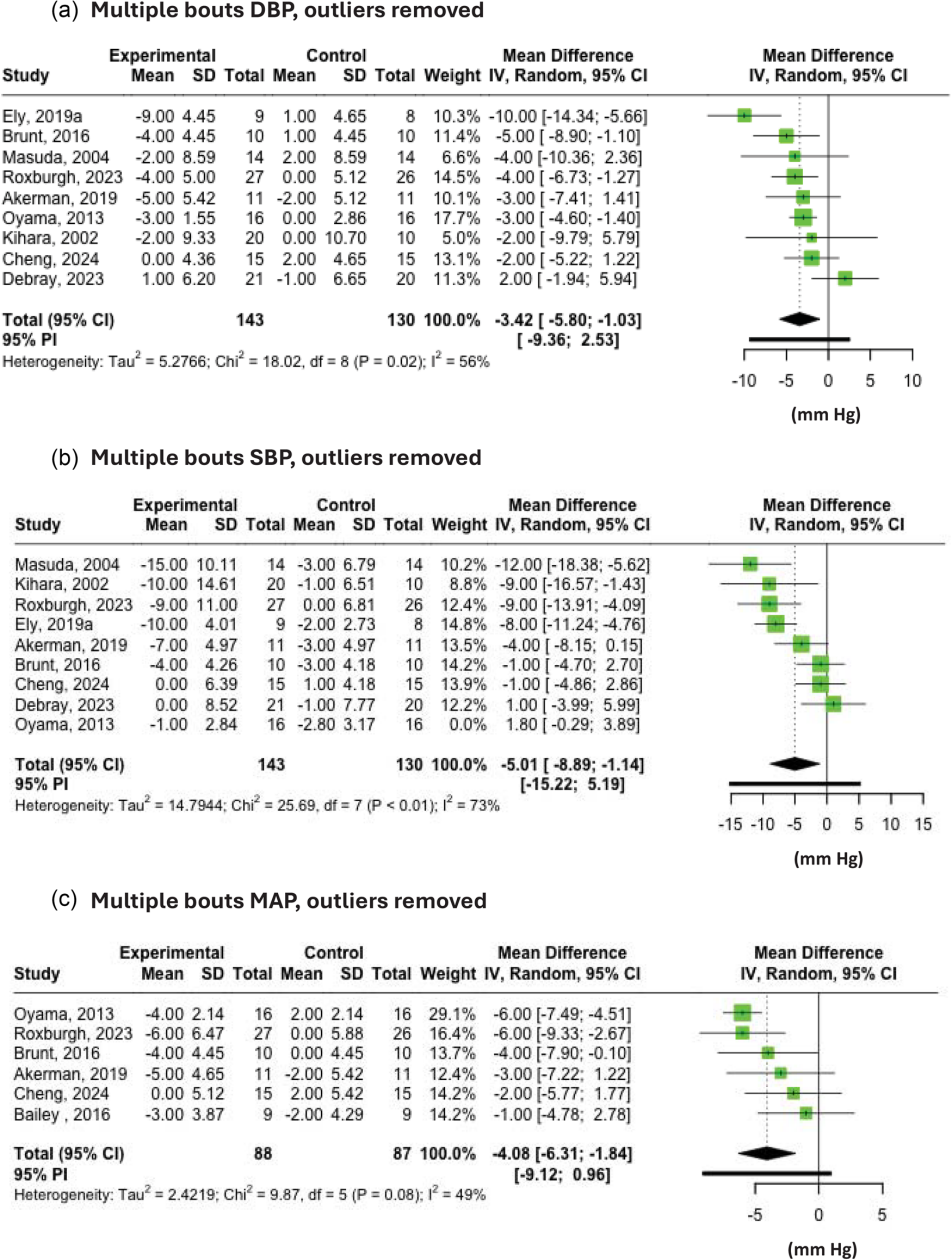
Pooled effect estimate of blood pressure response to multiple bouts of heat thermotherapy (HT) after removal of publication outliers. (a) Diastolic blood pressure (DBP) response to HT multiple bouts; (b) systolic blood pressure (SBP) response to HT multiple bouts; (c) mean arterial pressure (MAP) response to HT multiple bouts. Mean differences in the delta change (pre–post) between the intervention and control arms are presented in mmHg.

**FIGURE 4:**
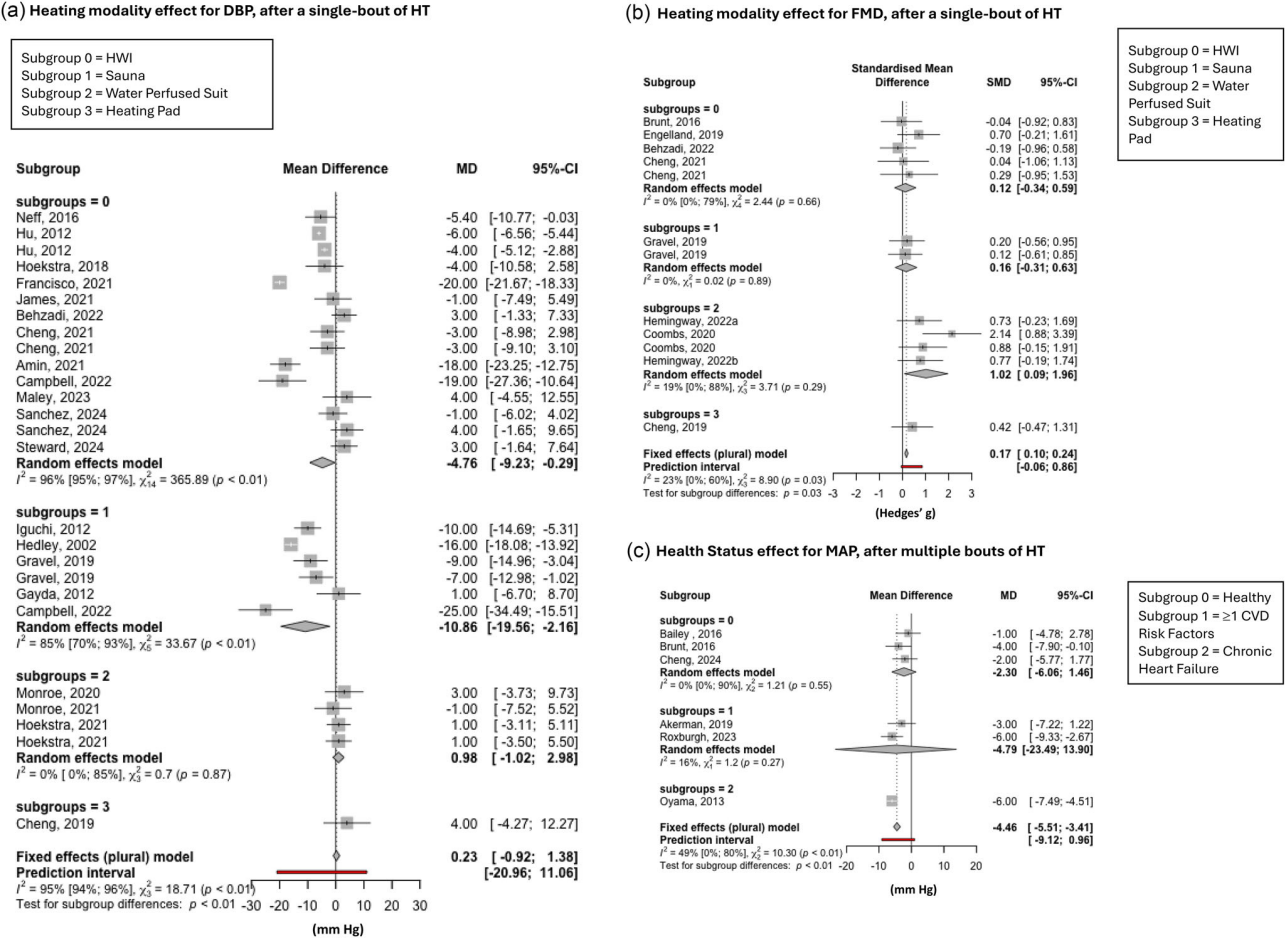
Subgroup effects of heating modality on diastolic blood pressure (DBP) and flow‐mediated dilation (FMD) following a single‐bout of heat thermotherapy (HT), and the effect of participant health status on mean arterial pressure (MAP). (a) DBP response across heating modalities; 0 = HWI; 1 = Sauna; 2 = Water Perfused Suit; 3 = heating pad. (b) FMD response across heating modalities; 0 = HWI; 1 = Sauna; 2 = Water Perfused Suit. (c) MAP response across different participant health status; 0 = healthy (no cardiovascular disease (CVD) risk factors; 1 = ≥1 CVD risk factor; 2 = chronic heart failure. Mean differences in the delta change (pre–post) between the intervention and control arms are presented in mmHg for blood pressure. Standardised mean differences in the delta change (pre–post) between the intervention and control arms are presented as an effect size (Hedges’ *g*) for FMD responses.

Mean arterial pressure (MAP) was significantly lowered following single and multiple HT bouts, as was diastolic blood pressure (DBP; Figures 2 and 3). Systolic blood pressure (SBP) did not change following a single HT bout but did after multiple bouts (Figures 2 and 3). There was a significant subgroup effect for heating modality on DBP following a single HT bout. DBP was lowest in the sauna group, followed by HWI (Figure 4a). There was a subgroup effect of health status on MAP after multiple HT bouts; however, there was no significant difference between healthy participants and those with CVD risk factors (Figure 4c).

**FIGURE 5:**
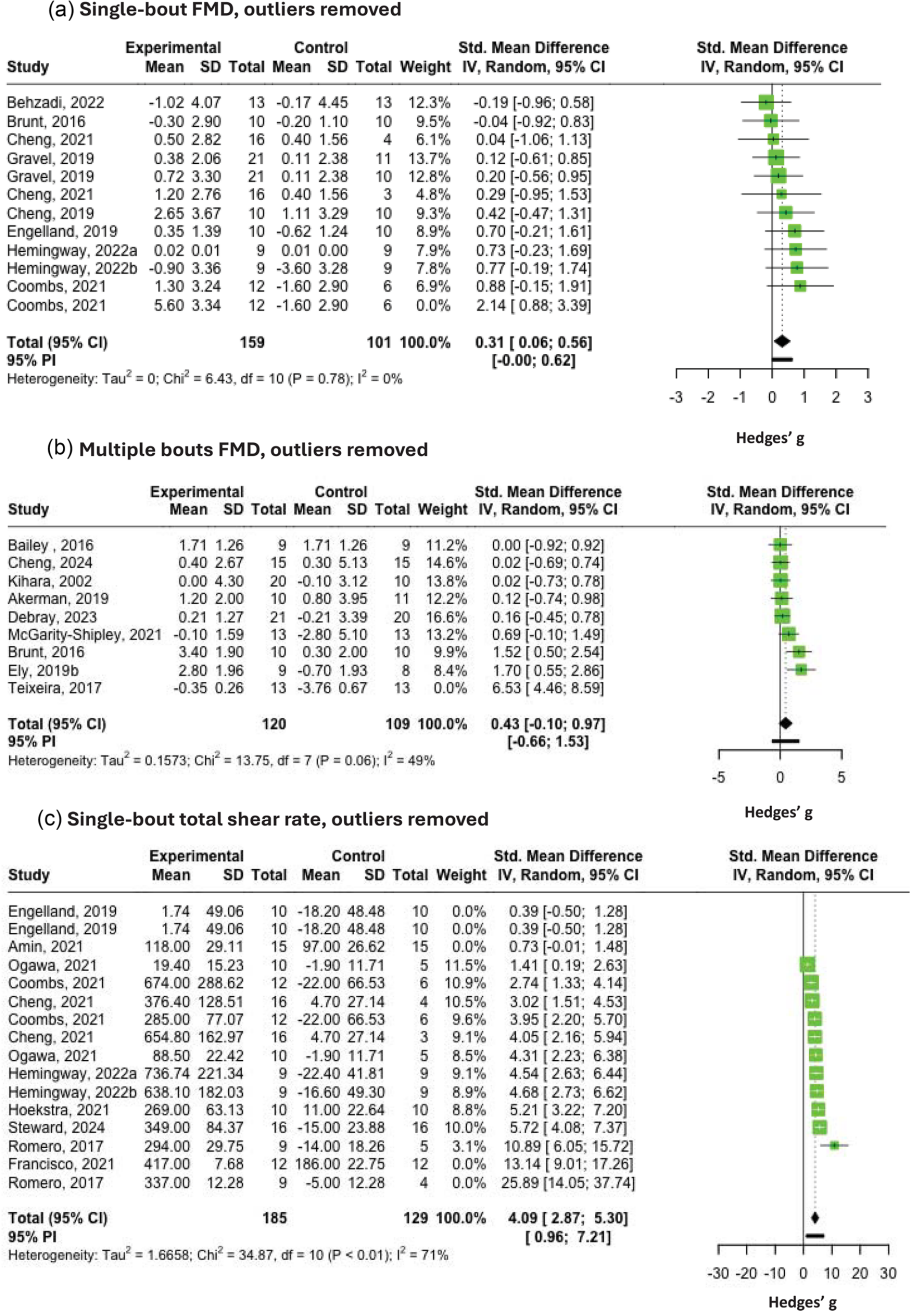
Pooled effect estimates of flow‐mediated dilation (FMD) to single and multiple bouts of heat thermotherapy (HT), and shear rate response to a single‐bout of HT after removal of publication outliers. (a) FMD response to HT single‐bout. (b) FMD response to HT multiple bouts. (c) Total shear rate response to an HT single bout. Standardised mean differences in the delta change (pre–post) between the intervention and control arms are presented as an effect size (Hedges’ *g*) for FMD and shear rate responses.

FMD and peripheral artery shear rate (Figure 5) significantly improved following a single‐bout of HT, but this did not persist with multiple bouts. There was no significant difference for arterial stiffness (*P* = 0.053; Hedges’ *g* = −0.43 [−0.86 to 0.01]). IL‐6 decreased following multiple bouts of HT, although the number of publications was limited (*n *= 2). There was no significant difference in glucose, HSP or CRP, potentially due to the small number of publications included for those variables. Finally, there was no significant modulating effect of cumulative minutes (log‐transformed) on the treatment effect for any CV or cardiometabolic parameter. Forest plots that are not presented in the manuscript are available in the Supporting information.

The heterogeneity scores for most CV and cardiometabolic parameters were substantial despite removing outliers (*I* ^2^ > 75%). Only HT single‐bout FMD and HT multiple‐bouts MAP achieved low‐to‐moderate levels of heterogeneity (*I* ^2^ < 50%). Despite conducting Baujat diagnostics (Baujat et al., 2002), leave one out analysis and DFFITS analysis (Cohen, 2013) to identify and remove outliers, this did not change the heterogeneity. Most of the funnel plots for the CV or cardiometabolic parameters displayed symmetry, and none of the Egger tests were significant (*P *> 0.05). These tests demonstrate that the meta‐analysis was unlikely to be influenced by publication and small study bias. The *p*‐curve analysis (Simonsohn et al., 2014) demonstrated a skew to the left for all meta‐analysis variables (most publications that were significant were <0.02 rather than close to 0.05). Therefore, meta‐analysis variables are unlikely to have publications with selective statistical reporting, for example where authors increase the number of participants until a *P*‐value of 0.05 is achieved (Simonsohn et al., 2014) or only selectively report significant *p*‐values.

Overall, included publications were deemed to have a low risk (*n* = 4), some concerns (*n *= 41) or a high risk of bias (*n *= 6). The main concern was the randomisation process, of which six publications were rated as high risk (*n *= 6) or some concerns (*n *= 41). The lowest area of concern was the risk of bias due to deviations from the intended interventions, in which all 51 publications were deemed to have a low risk of bias.

## 4 DISCUSSION

This systematic review and meta‐analysis, including 51 publications and 1055 participants, evaluated the efficacy of HT by comparing CV and cardiometabolic responses to acute versus multiple HT bouts. A single‐bout of HT acutely reduced DBP and MAP and significantly increased peripheral artery shear rate and FMD post‐HT. Multiple‐bouts of HT significantly reduced SBP, DBP and MAP. Participants’ age or health status did not significantly influence CV responses to HT. Thus, HT elicits beneficial acute CV responses and improves BP long‐term, irrespective of an individual's health status. However, the meta‐regression analysis was unable to identify the most effective HT strategy (i.e. heating modality, duration of HT bout) for eliciting positive CV and cardiometabolic responses, likely due to substantial subgroup heterogeneity across publications.

### 4.1 Blood pressure

To our knowledge, this is the first meta‐analysis to examine CV function following a single HT bout. The findings demonstrate that, irrespective of individuals’ health status, both single and multiple bouts of HT significantly lowered MAP and DBP, indicating HT had a consistent hypotensive effect across different HT exposures. In contrast, SBP did not change following a single HT bout but was significantly reduced after multiple exposures. Both DBP and SBP hypertension independently contribute to the risk of adverse CVD events, with a 2 mmHg reduction in DBP and a 10 mmHg reduction in SBP each associated with a lower risk of such events (Ettehad et al., 2016; Flint et al., 2019). The current meta‐analysis indicates that HT has the potential to elicit clinically meaningful reductions in DBP. Although significant reductions in SBP can also be achieved following multiple HT bouts, the clinical relevance of HT for this parameter remains uncertain.

The type of HT modality influenced the magnitude of DBP reduction following a single HT bout, with sauna bathing causing the largest decrease, followed by HWI (−11 vs −5 mmHg; Figure 4). This may reflect differences in thermal load, exposure duration and/or physiological responses between HT modalities. Notably, the hydrostatic effect of water immersion has been shown to increase venous return and intracardiac pressures, thereby helping to maintain stroke volume during HWI despite thermoregulatory and CV strain (Francisco et al., 2021; Tei et al., 1995). Nevertheless, HWI has also been shown to cause a similar or slightly larger hypotensive effect (driven by a drop in systemic vascular resistance and DBP) both during and post WI, when directly compared to exercise (matched for time and *T*_c_ rise; Francisco et al., 2021) or sauna (albeit a shorter duration and more rapid *T*_c_ rise in sauna vs HWI; Campbell et al., 2022). Thus, other factors may have contributed to the larger DBP reduction observed in the current meta‐analysis with sauna bathing. Given the heterogeneity of HT strategies used across the included publications, this heat modality subgroup analysis should be interpreted with caution. Although a subgroup effect of health status on MAP was observed after multiple HT bouts, no statistically significant difference was found between healthy individuals and those with CV risk factors. This implies that HT may be broadly effective across populations, although again, the lack of significance may also reflect limited sample sizes or study heterogeneity.

BP responses are influenced by mild changes in air temperature (Lanzinger et al., 2014) and routine movements, such as lying or standing (Lucas et al., 2010). In the current meta‐analysis, the included publications did not consistently report participant state (e.g. postural position) or environmental conditions (e.g. air temperature). Moreover, it remains unclear whether all the included publications adhered to established BP measurement guidelines (Stergiou et al., 2021). Future research should aim to standardise and clearly report these methodological factors.

### 4.2 Flow‐mediated dilation and shear rate

This meta‐analysis identified significant improvements in FMD and peripheral artery shear rate following a single HT bout, indicating acute enhancements in endothelial function and vascular responsiveness following HT. These increases in shear rate are expected, as an elevated *T*_c_ drives a redistribution of blood flow to the periphery (Rowell, 1974), increasing antegrade flow and reducing retrograde flow, mechanisms known to influence FMD (Carter et al., 2013; Francisco et al., 2021; Tinken et al., 2009).

The magnitude of HT‐related shear rate and FMD responses appears highly dependent on the timing of post‐HT measurements. Previous studies have reported shear rate and FMD changes when measured 10–45 min post‐HT (Cheng et al., 2021; Coombs et al., 2021; Romero et al., 2017; Tinken et al., 2009), while others found no change with measurements 40–60 min post‐HT (Behzadi et al., 2022; Brunt, Jeckell et al., 2016; Engelland et al., 2019). Thus, acute HT‐related FMD responses appear to be short‐lasting, resolving within 30 min. Moreover, limb versus whole‐body heating can differentially influence FMD, with limb heating improving FMD whereas whole‐body heating acutely attenuates it—due to increased baseline diameter, reduced shear stimulus, and heightened sympathetic activation associated with whole‐body heating (Chaseling et al., 2023). Across included publications, variation in measurement timing and the use of whole‐body versus partial‐body heating likely contributed to the heterogeneity in effect sizes observed in this meta‐analysis, reflecting broader methodological inconsistencies within the current HT literature. These methodological differences may also explain the absence of significant FMD changes following multiple bouts of HT. Further research is needed to clarify the chronic effects of HT, particularly in populations who may benefit most from alternative strategies, such as those who are unable or unwilling to exercise or are resistant to pharmacological treatment.

### 4.3 Cardiometabolic health

Based on the current meta‐analysis, it is unclear whether HT improves cardiometabolic health. HT was shown to cause a significant long‐term reduction in IL‐6; however, this finding is based on just two publications. Due to the lack of publications, some cardiometabolic variables were pooled (e.g. fasting and postprandial glucose values were pooled, as were HSP), which increased the heterogeneity of our analysis; further research in this area is warranted.

### 4.4 Meta‐regression

The meta‐regression did not reveal the most effective HT strategy (i.e. heating modality, duration of HT bout) for eliciting positive CV and cardiometabolic responses. Substantial heterogeneity was observed in both participant characteristics and protocol designs for each variable. This was due to the limited number of eligible publications, which necessitated pooling all included studies (both single and multiple HT sessions).

### 4.5 Strengths and limitations

This systematic review and meta‐analysis is the most comprehensive assessment of HT literature to date, including 51 controlled studies, which enhances confidence in reported HT outcomes/comparisons. Uniquely, it distinguishes between single and multiple HT bouts, allowing for a systematic assessment of both acute responses and chronic adaptations in cardiovascular and cardiometabolic parameters.

Substantial heterogeneity (*I* ^2^ > 75%) was observed for most CV and cardiometabolic variables, likely due to protocol differences across publications. Subgroup analyses (i.e. heating modality) were conducted to explore these differences, but did not resolve the heterogeneity. Some subgroup analyses were limited by a small number of publications (< 10) and the presence of outliers, which may have skewed the results. Due to limited data availability, mechanistically distinct variables (e.g. glucose measures and HSP isoforms) were grouped, which may obscure specific physiological responses and assumes a shared directional response to heat therapy.

The risk of bias assessment showed that most publications (47/51) had some concerns or a high risk of bias. This was mainly due to inadequate reporting of randomisation methods or the use of a matched pairs design, which can introduce selection bias. Additionally, the lack of pre‐registered studies raised concerns about selective reporting. Overall, these findings suggest that HT research remains at the proof‐of‐concept stage.

This review and meta‐analysis aimed to examine how moderating factors, such as heating modality and age, affected CV and cardiometabolic responses to single and multiple HT sessions. To maximise the number of eligible studies, core body temperature (*T*_c_) and hydration status did not form part of the inclusion criteria, despite the well‐established influence of heat strain (i.e. elevated *T*_c_) and dehydration on CV and cardiometabolic outcomes (Crandall & González‐Alonso, 2010; Rowell, 1974). Due to the limited reporting of *T*_c_ in the included studies, a direct assessment of heat strain was not possible. Instead, a meta‐regression was employed to examine the relationship between cumulative HT duration and CV and cardiometabolic parameters.

During screening, reviewers (B.P. and R.G.) randomly cross‐checked 20% of each other's publications rather than double‐screening all records. While this may have introduced bias or reduced the number of included studies (Stoll et al., 2019), reviewer agreement was high, suggesting a low risk of error (McDonagh et al., 2013). Additionally, a third reviewer (A.A.) conducted spot checks on extracted data.

### 4.6 Future research recommendations

Several parameters (e.g. CRP [multiple bouts]) were underpowered (<10 publications included). Therefore, it remains unclear whether HT improves these parameters. At this stage, public guidelines for HT cannot be established, nor can its effectiveness for CV or cardiometabolic health be confirmed. Future research should refine HT protocols to identify the optimal and minimal conditions needed for health benefits. Improved reporting is also essential, including HT‐induced changes in *T*_c_, post‐HT environmental conditions, participant hydration status, timing of measurements, and adherence to data collection guidelines (e.g. BP measurement, female participants’ demographic information). This would help clarify underlying mechanisms, reduce protocol heterogeneity and strengthen confidence in reported outcomes.

### 4.7 Conclusion

This meta‐analysis provides novel insights into the CV and cardiometabolic effects of HT, particularly following a single bout. HT consistently reduced MAP and DBP across different modalities and populations, with sauna bathing producing the largest acute reductions in DBP. SBP reductions were only observed after multiple HT exposures, and the clinical relevance of these changes remains to be fully established. Acute improvements in endothelial function and shear rate following HT suggest transient vascular benefits. However, methodological inconsistencies (such as timing of measurements and heating modality) likely influenced the outcomes of this meta‐analysis. Overall, these findings support the potential of HT as a non‐pharmacological strategy to improve CV parameters. Further research is needed to better understand the potential of heat therapy to improve cardiometabolic parameters.

## AUTHOR CONTRIBUTIONS

Concept and design: Ben S. Price, Samuel J. E. Lucas, Ashley P. Akerman and Rebekah A. I. Lucas. Data acquisition, analysis and interpretation: Ben S. Price, Samuel J. E. Lucas, Ashley P. Akerman, Rachel E. Gilworth and Rebekah A. I. Lucas. All authors have read and approved the final version of this manuscript and agree to be accountable for all aspects of the work in ensuring that questions related to the accuracy or integrity of any part of the work are appropriately investigated and resolved. All persons designated as authors qualify for authorship, and all those who qualify for authorship are listed.

## CONFLICT OF INTEREST

The authors declare they have no conflicts of interest.

## Supporting information



Supporting Information

Supporting Information

## Data Availability

All data supporting the results of this study are available within the paper and its Supporting Information files. Additional raw data can be obtained from the corresponding author upon reasonable request.
