# Supplementary material for: Heat thermotherapy to improve cardiovascular function and cardiometabolic health: A systematic review and meta‐analysis
Source: Exp Physiol. 2025 Oct 30:10.1113/EP092404. Online ahead of print. doi: 10.1113/EP092404 (PMC13394758; doi:10.1113/EP092404)
Supplement: Supplementary file 1 — Supporting Information [file EPH-9999-0-s002.pptx]

## Slide 1
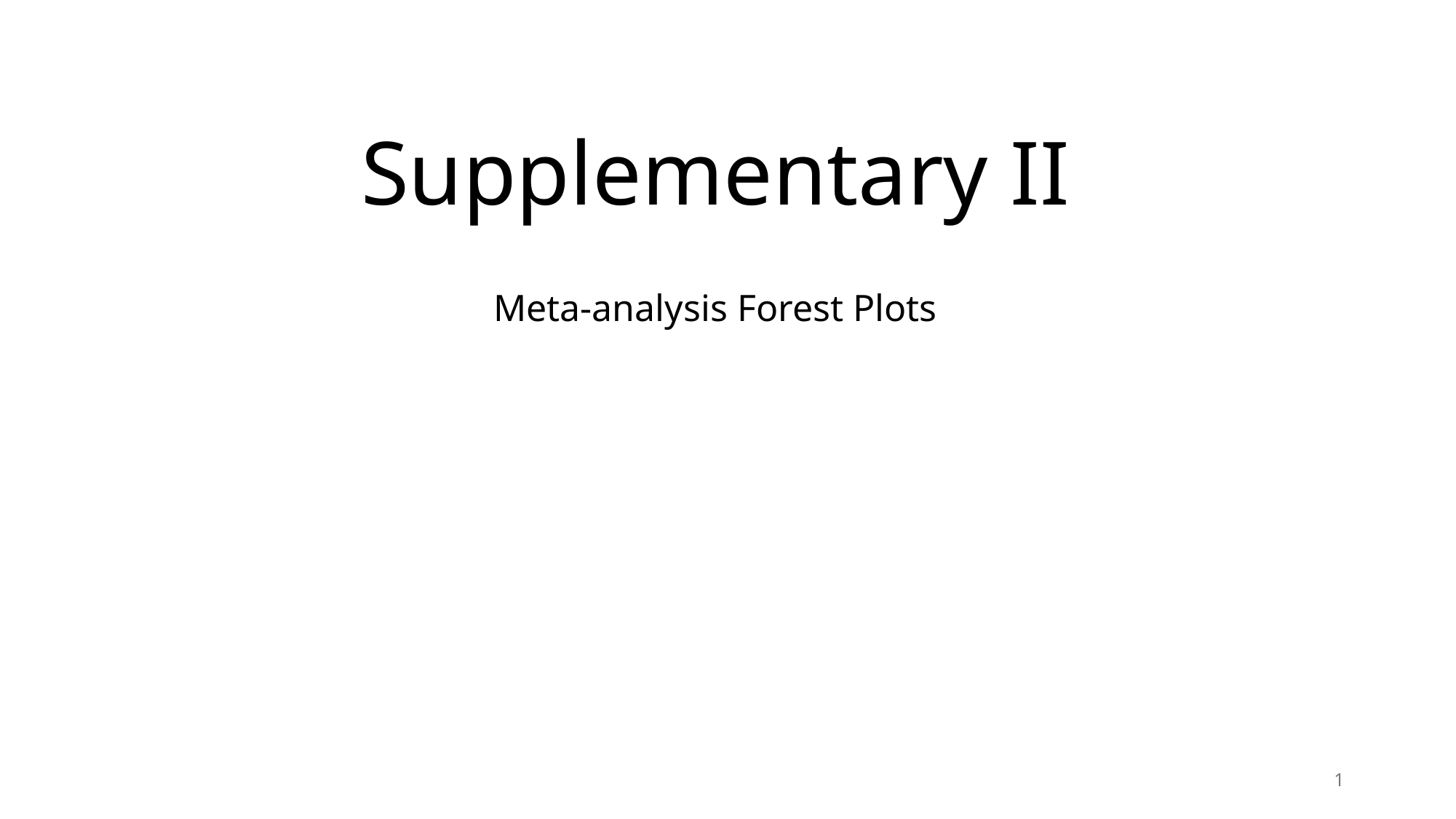

# Supplementary II
Meta-analysis Forest Plots
1

## Slide 2
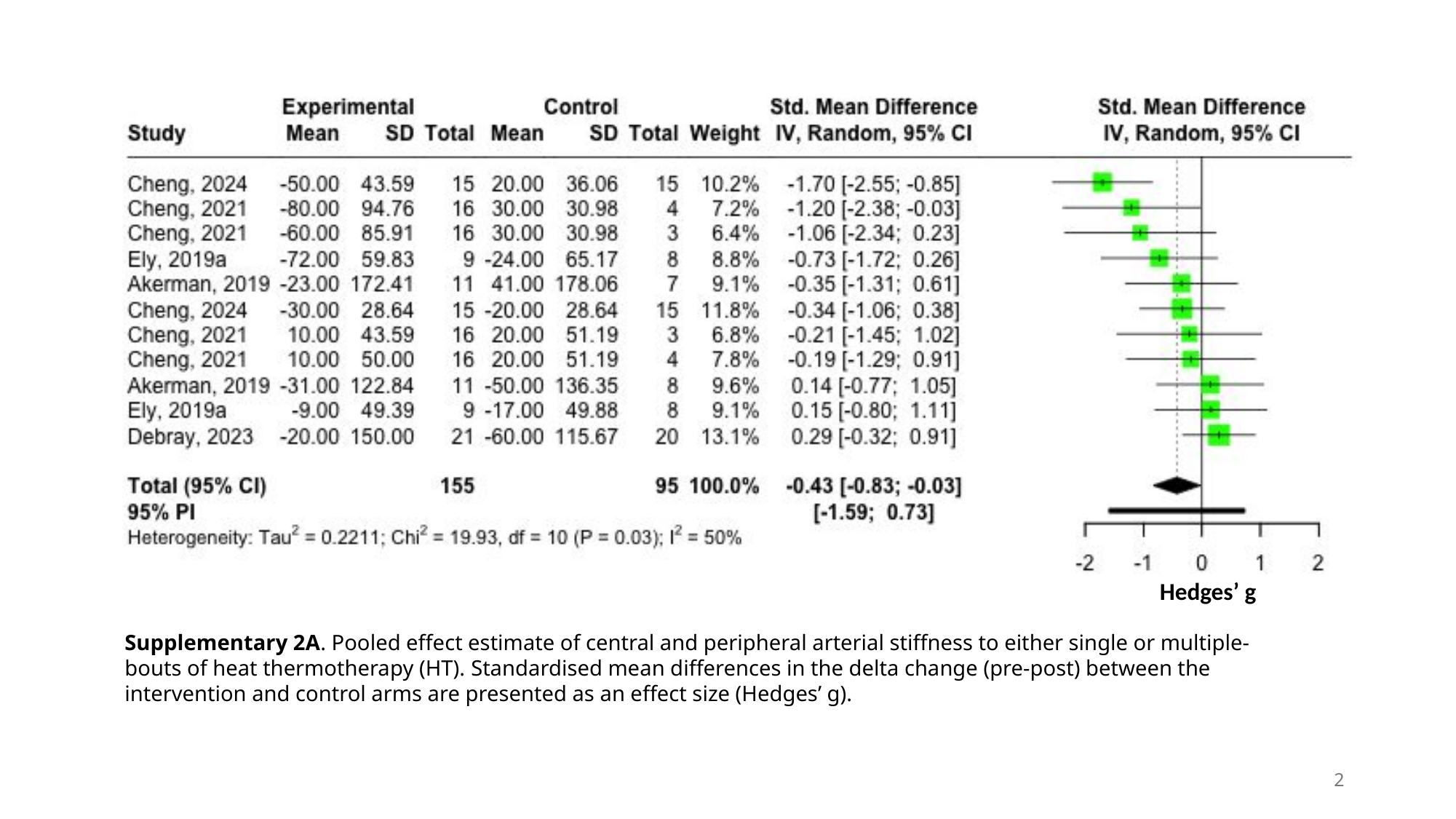

Hedges’ g
Supplementary 2A. Pooled effect estimate of central and peripheral arterial stiffness to either single or multiple-bouts of heat thermotherapy (HT). Standardised mean differences in the delta change (pre-post) between the intervention and control arms are presented as an effect size (Hedges’ g).
2

## Slide 3
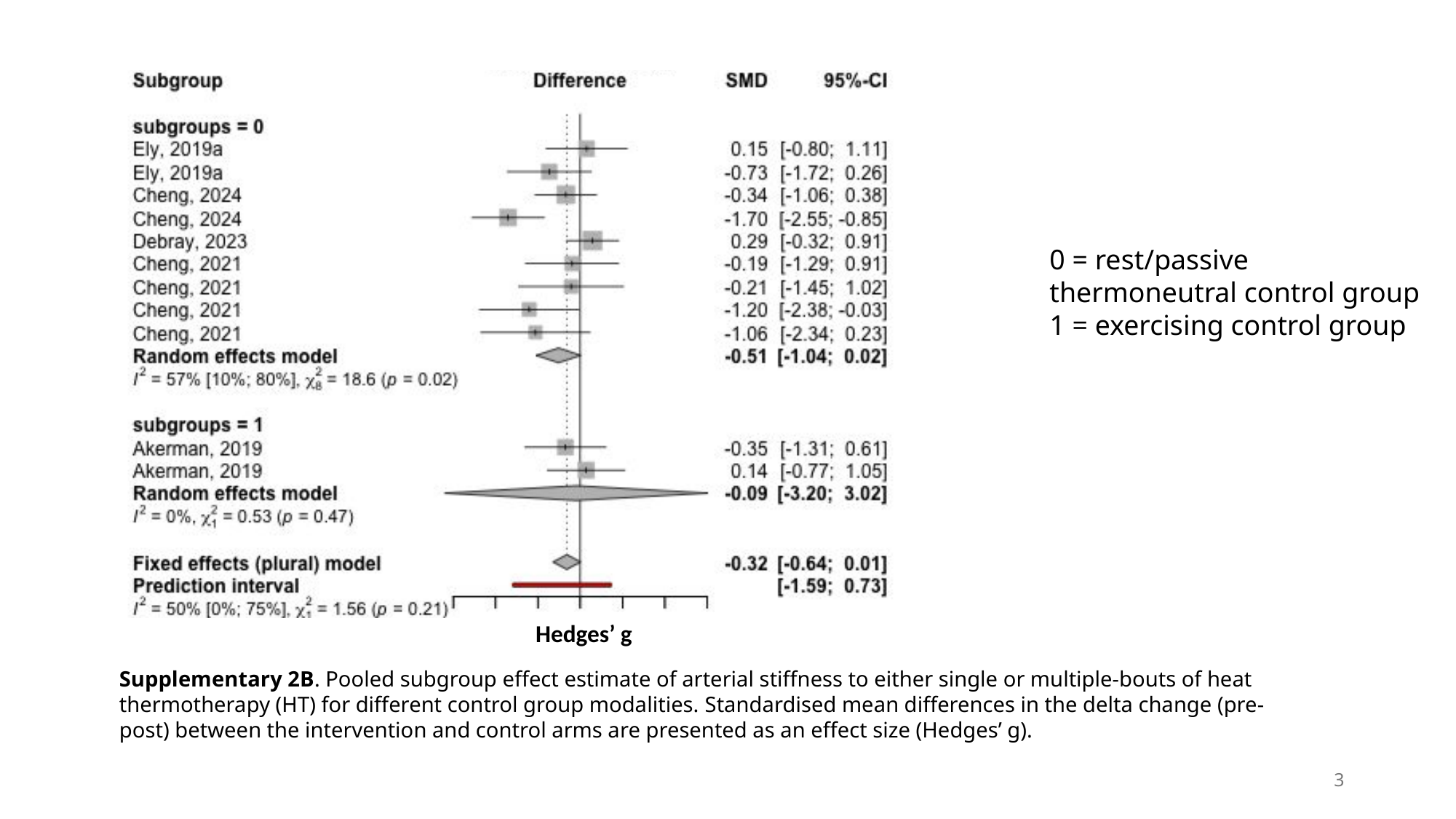

0 = rest/passive thermoneutral control group
1 = exercising control group
Hedges’ g
Supplementary 2B. Pooled subgroup effect estimate of arterial stiffness to either single or multiple-bouts of heat thermotherapy (HT) for different control group modalities. Standardised mean differences in the delta change (pre-post) between the intervention and control arms are presented as an effect size (Hedges’ g).
3

## Slide 4
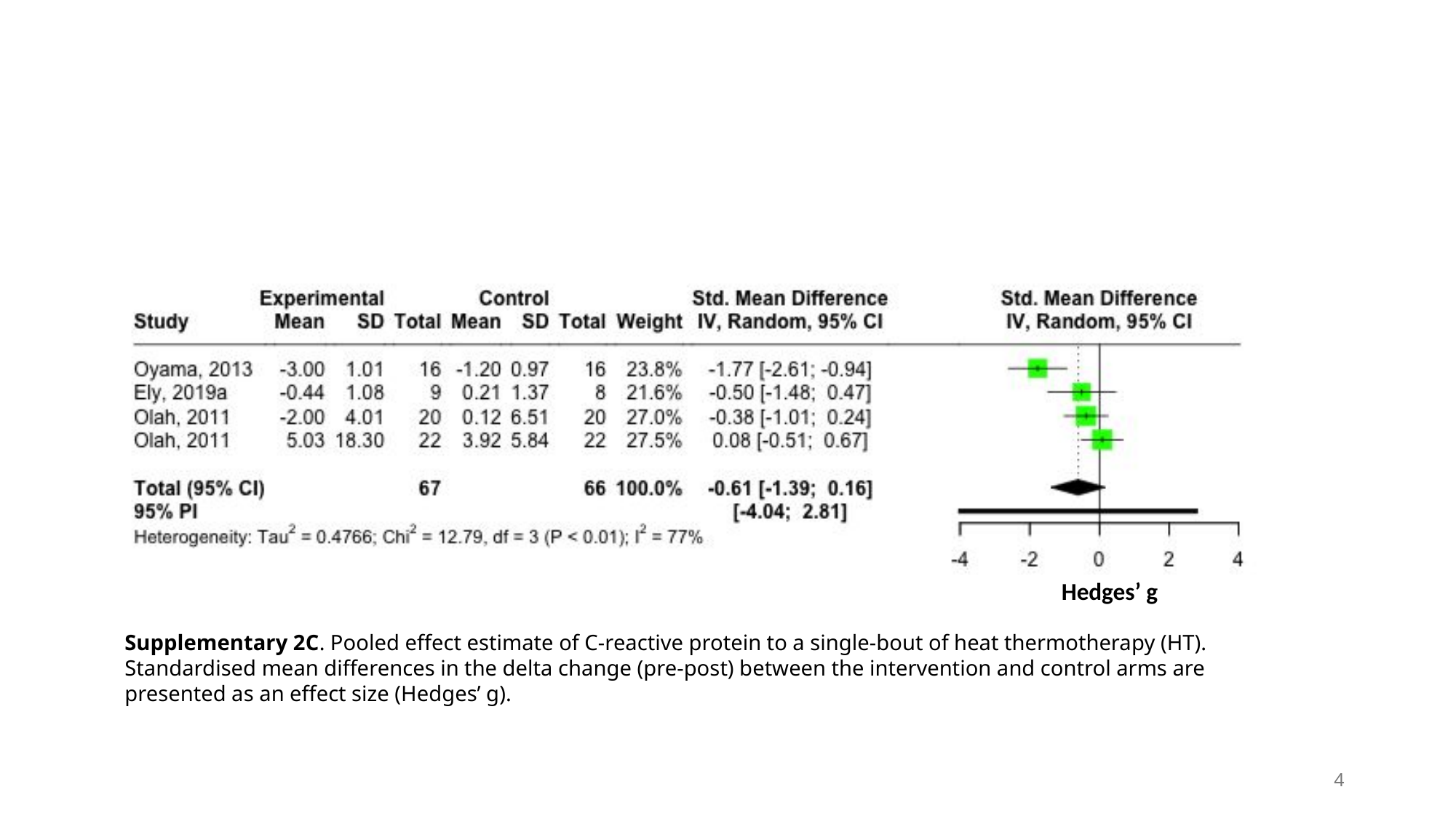

Hedges’ g
Supplementary 2C. Pooled effect estimate of C-reactive protein to a single-bout of heat thermotherapy (HT). Standardised mean differences in the delta change (pre-post) between the intervention and control arms are presented as an effect size (Hedges’ g).
4

## Slide 5
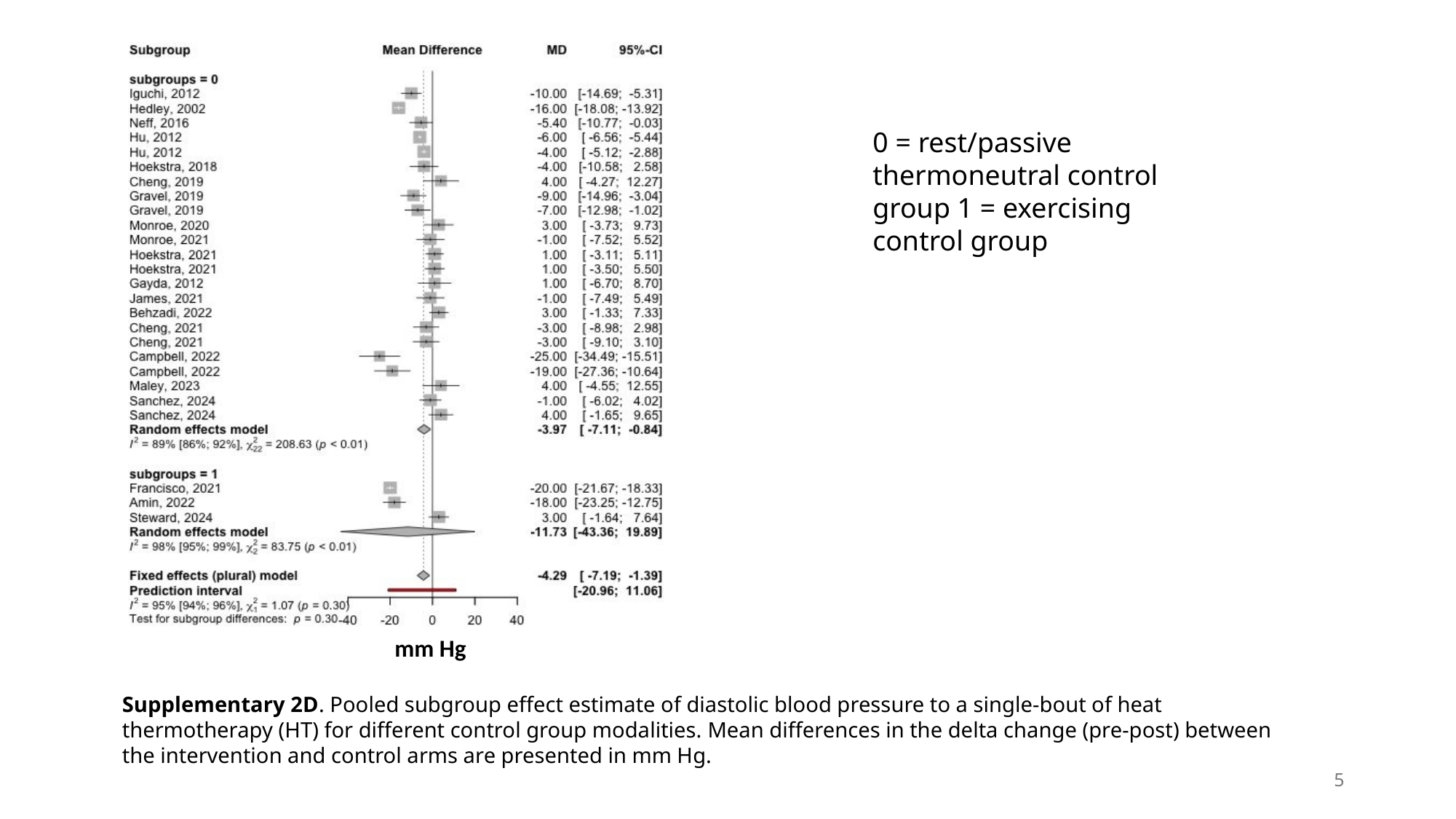

0 = rest/passive thermoneutral control group 1 = exercising control group
mm Hg
Supplementary 2D. Pooled subgroup effect estimate of diastolic blood pressure to a single-bout of heat thermotherapy (HT) for different control group modalities. Mean differences in the delta change (pre-post) between the intervention and control arms are presented in mm Hg.
5

## Slide 6
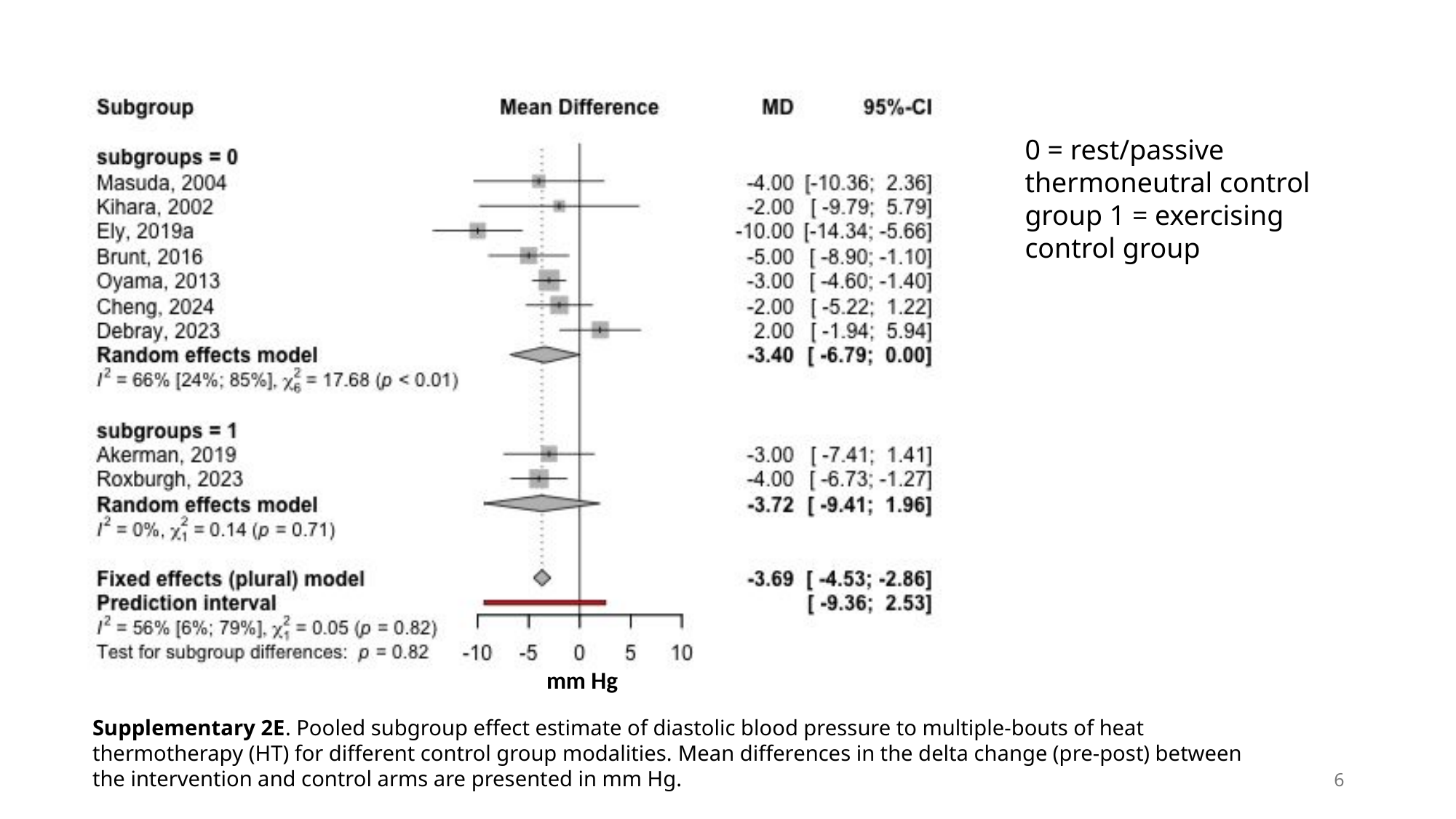

0 = rest/passive thermoneutral control group 1 = exercising control group
mm Hg
Supplementary 2E. Pooled subgroup effect estimate of diastolic blood pressure to multiple-bouts of heat thermotherapy (HT) for different control group modalities. Mean differences in the delta change (pre-post) between the intervention and control arms are presented in mm Hg.
6

## Slide 7
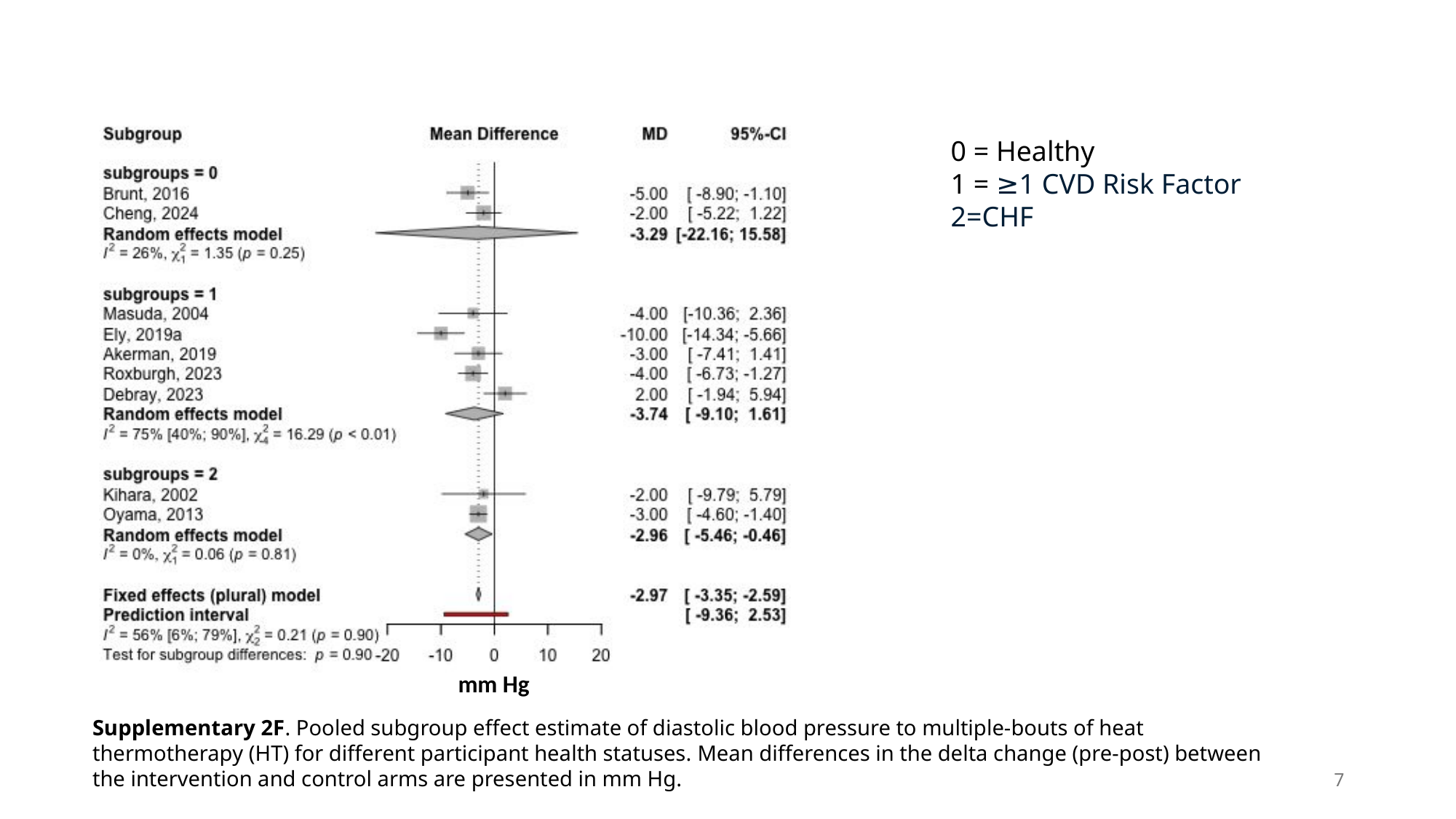

0 = Healthy
1 = ≥1 CVD Risk Factor
2=CHF
mm Hg
Supplementary 2F. Pooled subgroup effect estimate of diastolic blood pressure to multiple-bouts of heat thermotherapy (HT) for different participant health statuses. Mean differences in the delta change (pre-post) between the intervention and control arms are presented in mm Hg.
7

## Slide 8
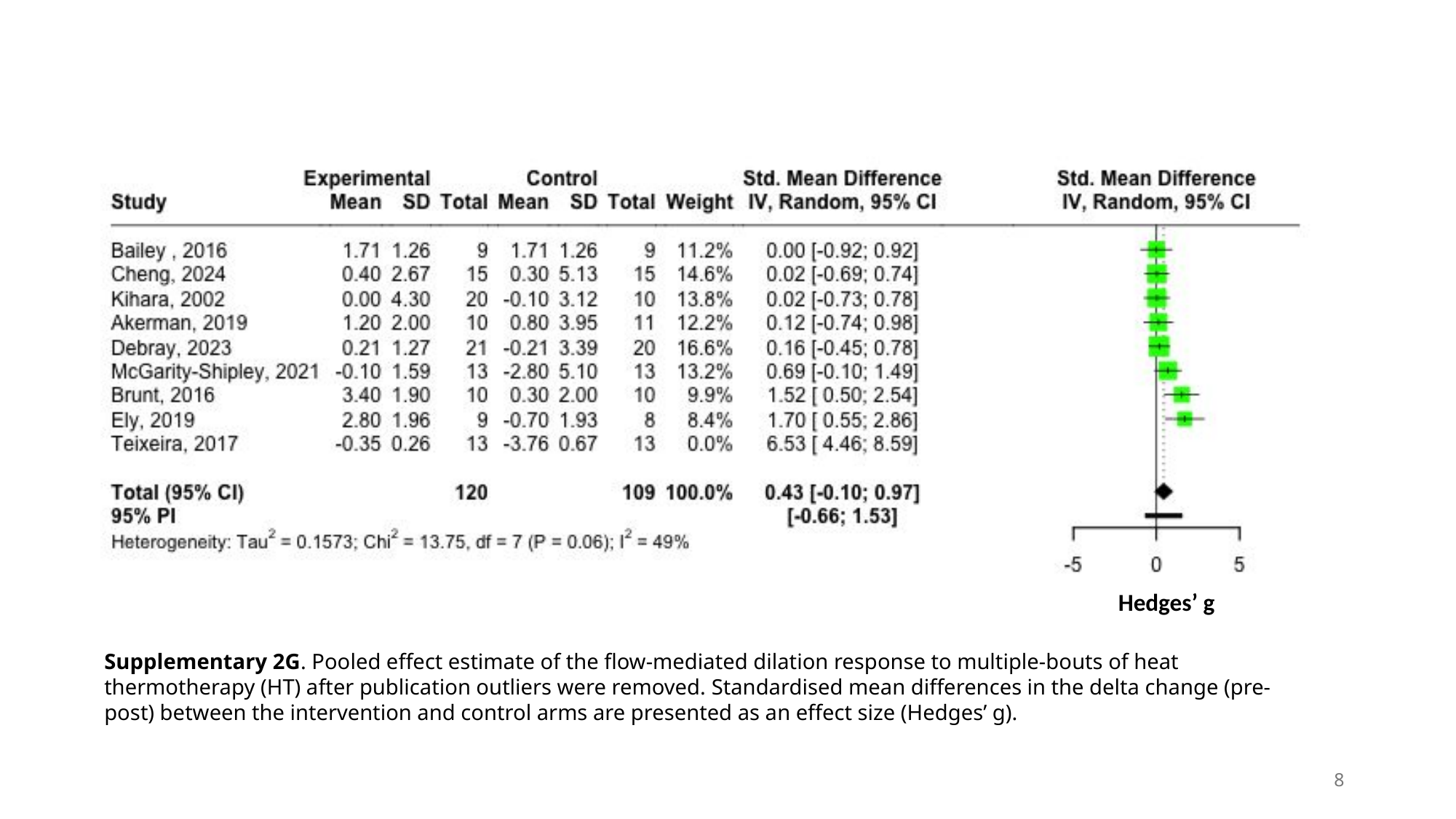

Hedges’ g
Supplementary 2G. Pooled effect estimate of the flow-mediated dilation response to multiple-bouts of heat thermotherapy (HT) after publication outliers were removed. Standardised mean differences in the delta change (pre-post) between the intervention and control arms are presented as an effect size (Hedges’ g).
8

## Slide 9
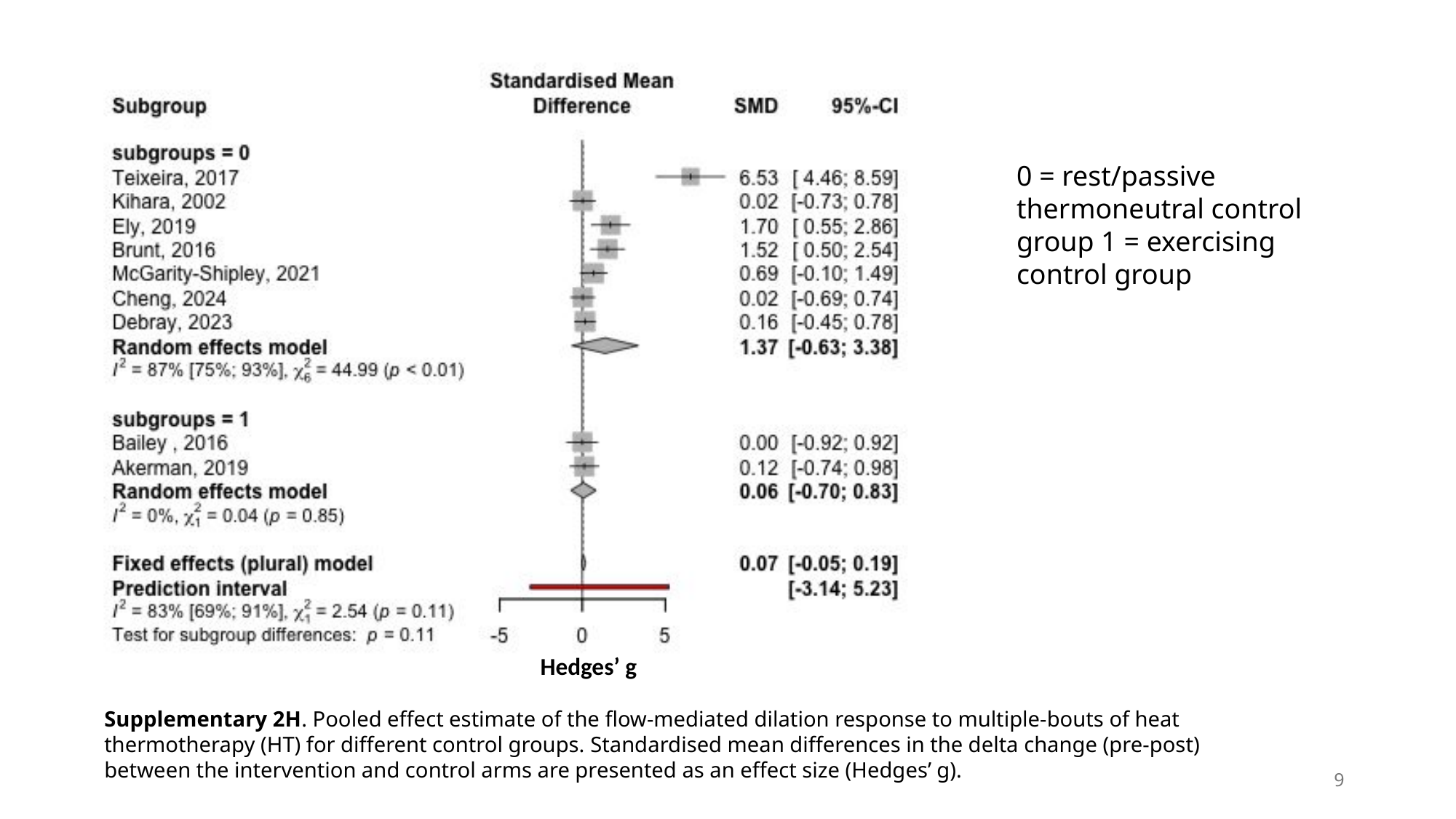

0 = rest/passive thermoneutral control group 1 = exercising control group
Hedges’ g
Supplementary 2H. Pooled effect estimate of the flow-mediated dilation response to multiple-bouts of heat thermotherapy (HT) for different control groups. Standardised mean differences in the delta change (pre-post) between the intervention and control arms are presented as an effect size (Hedges’ g).
9

## Slide 10
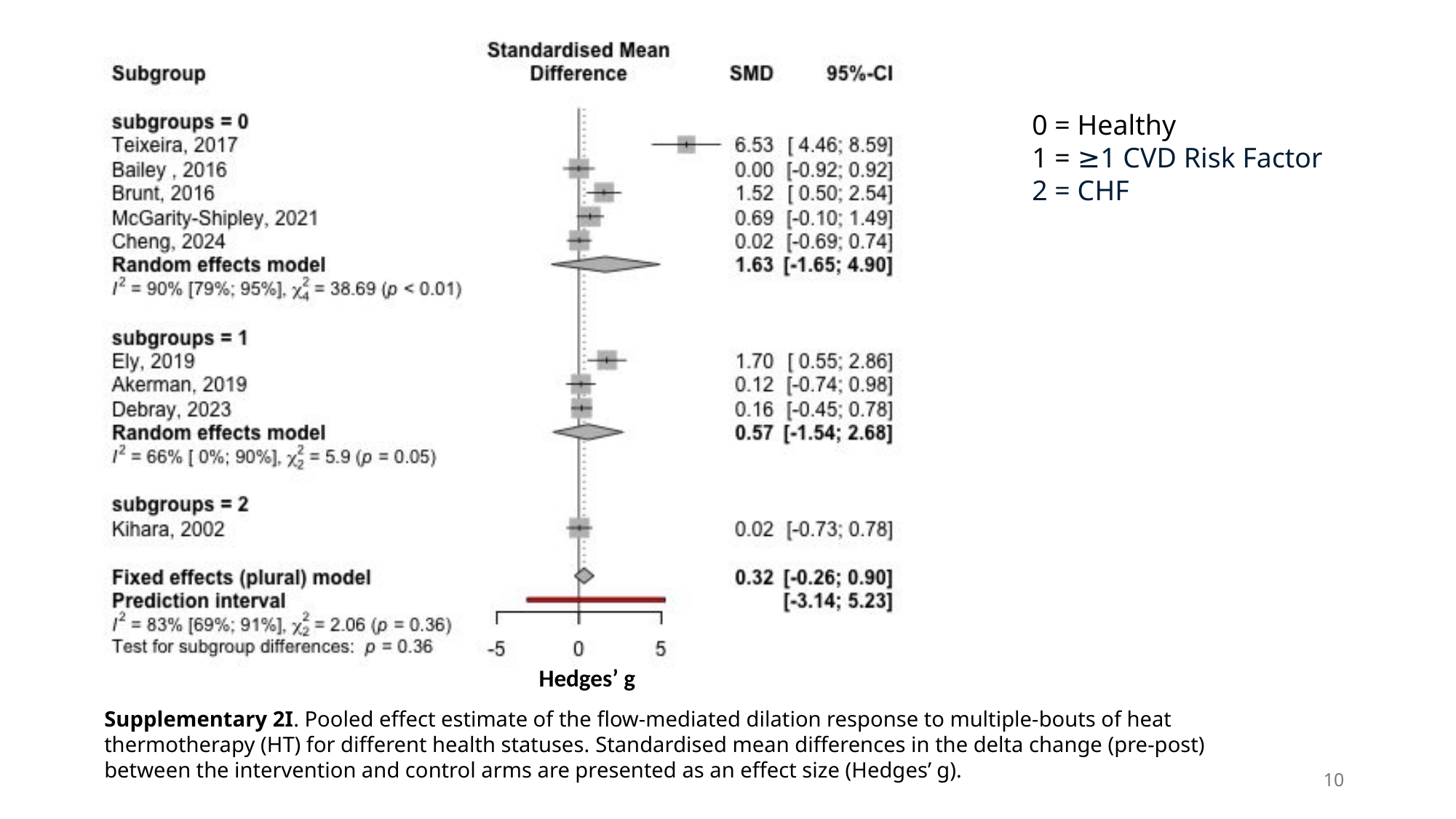

0 = Healthy
1 = ≥1 CVD Risk Factor
2 = CHF
Hedges’ g
Supplementary 2I. Pooled effect estimate of the flow-mediated dilation response to multiple-bouts of heat thermotherapy (HT) for different health statuses. Standardised mean differences in the delta change (pre-post) between the intervention and control arms are presented as an effect size (Hedges’ g).
10

## Slide 11
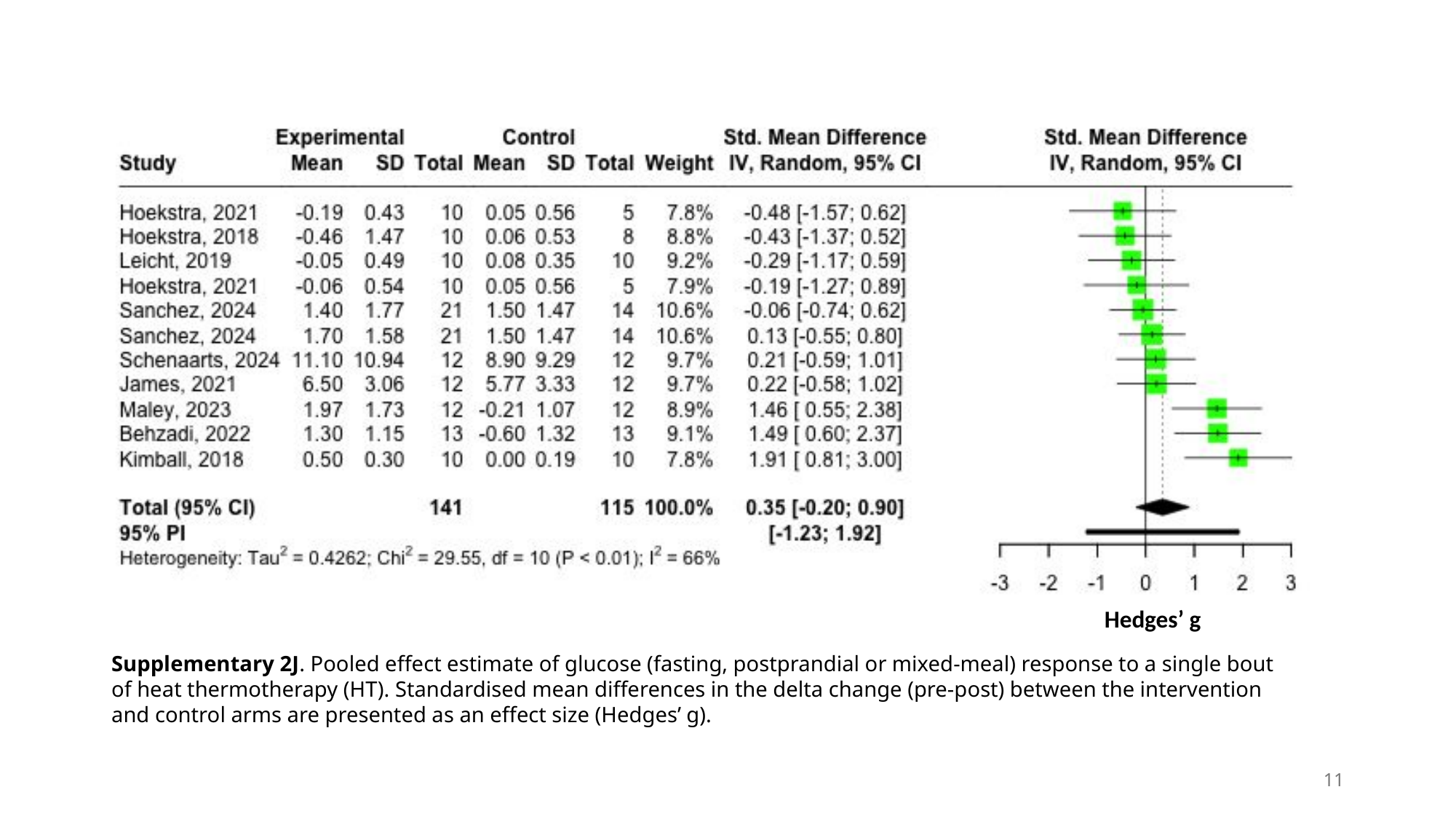

Hedges’ g
Supplementary 2J. Pooled effect estimate of glucose (fasting, postprandial or mixed-meal) response to a single bout of heat thermotherapy (HT). Standardised mean differences in the delta change (pre-post) between the intervention and control arms are presented as an effect size (Hedges’ g).
11

## Slide 12
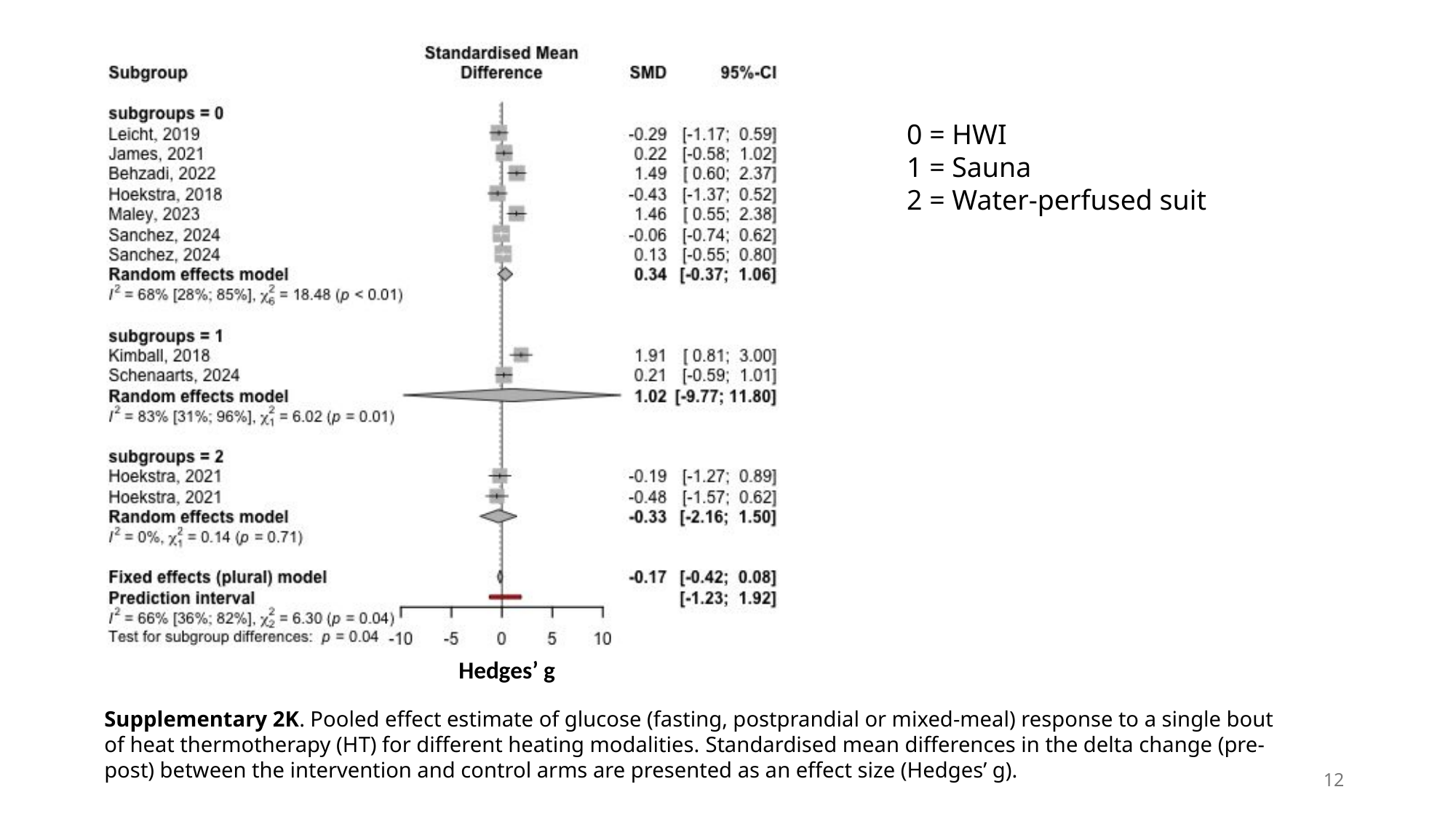

0 = HWI
1 = Sauna
2 = Water-perfused suit
Hedges’ g
Supplementary 2K. Pooled effect estimate of glucose (fasting, postprandial or mixed-meal) response to a single bout of heat thermotherapy (HT) for different heating modalities. Standardised mean differences in the delta change (pre-post) between the intervention and control arms are presented as an effect size (Hedges’ g).
12

## Slide 13
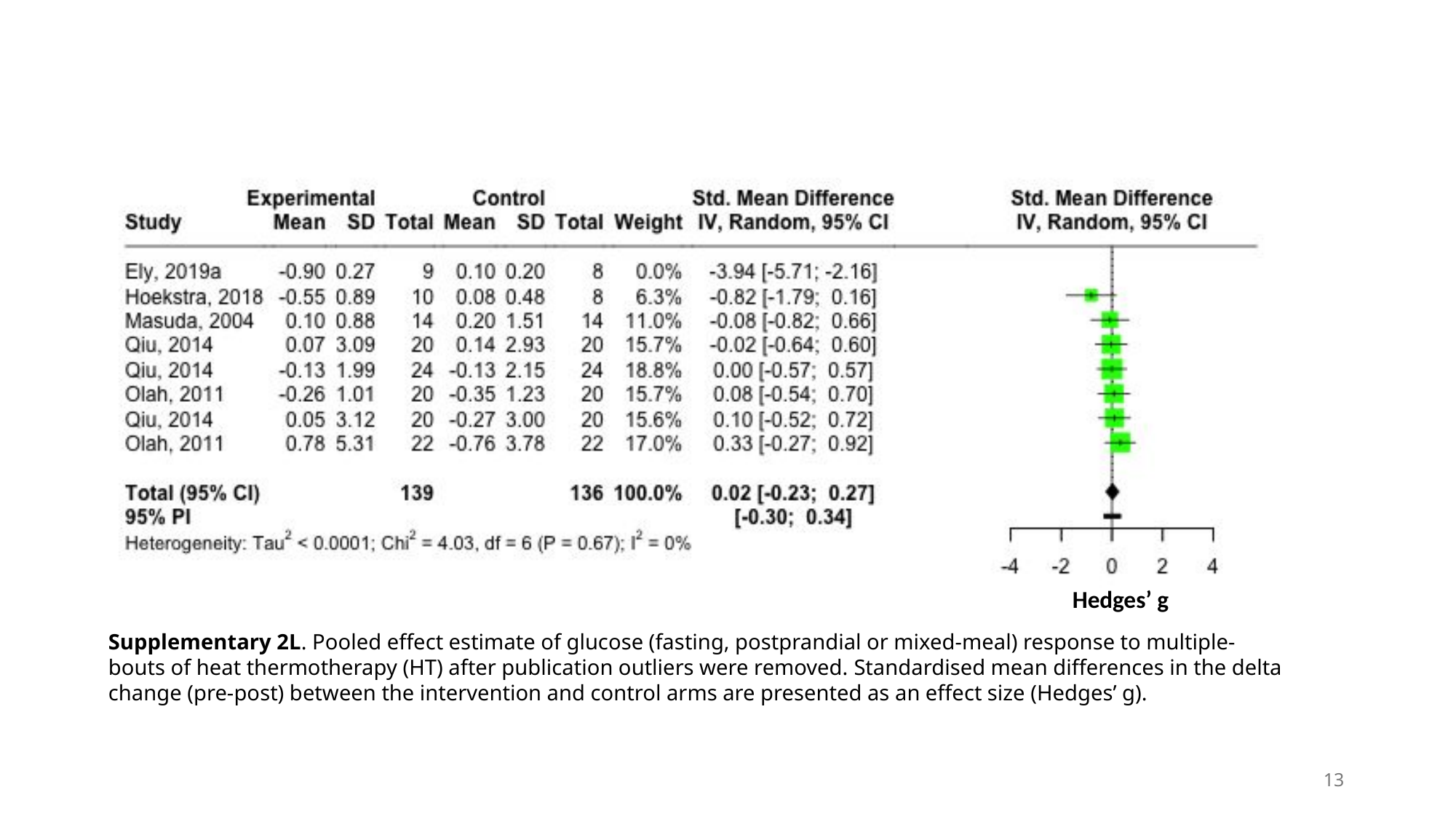

Hedges’ g
Supplementary 2L. Pooled effect estimate of glucose (fasting, postprandial or mixed-meal) response to multiple-bouts of heat thermotherapy (HT) after publication outliers were removed. Standardised mean differences in the delta change (pre-post) between the intervention and control arms are presented as an effect size (Hedges’ g).
13

## Slide 14
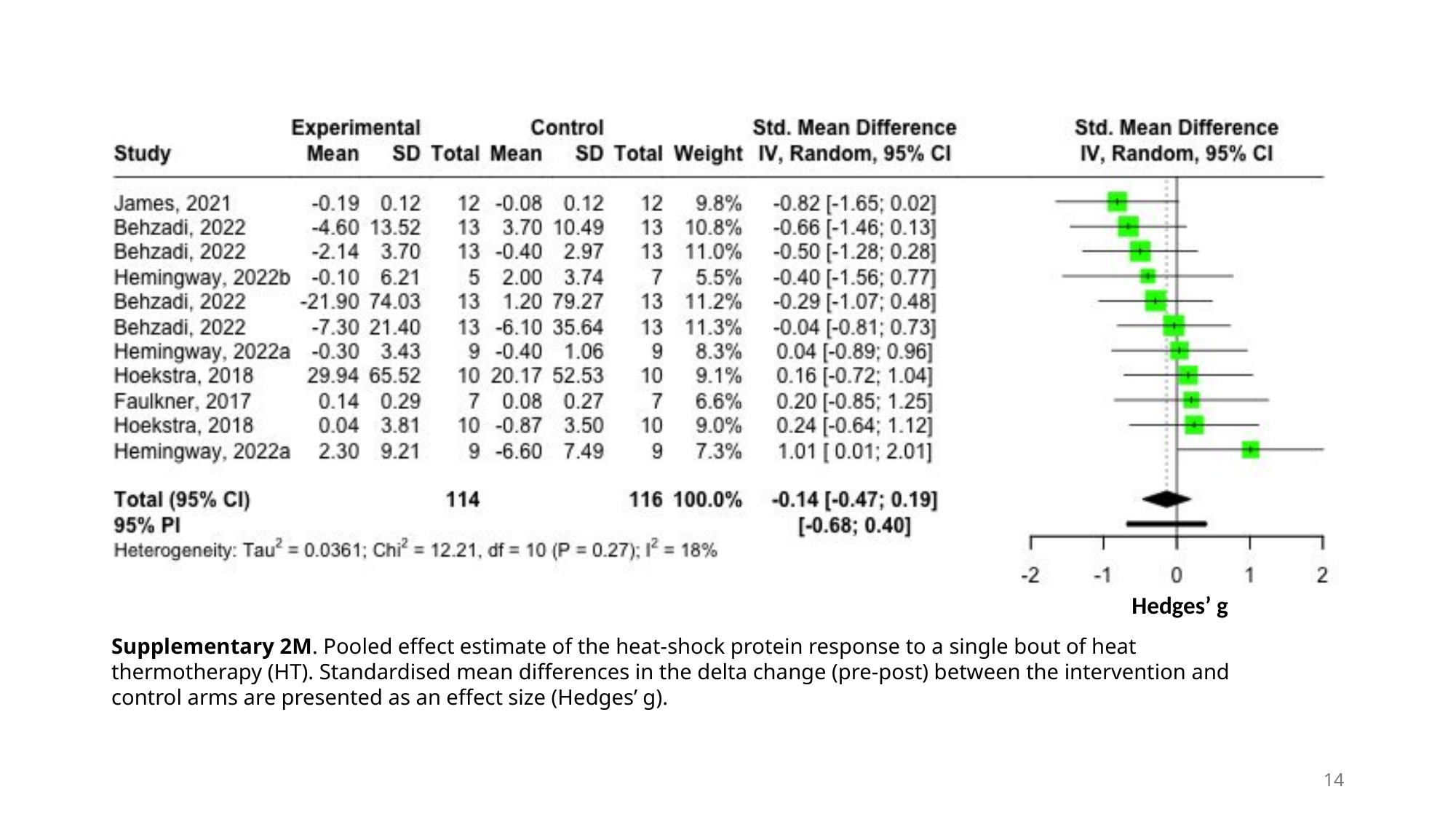

Hedges’ g
Supplementary 2M. Pooled effect estimate of the heat-shock protein response to a single bout of heat thermotherapy (HT). Standardised mean differences in the delta change (pre-post) between the intervention and control arms are presented as an effect size (Hedges’ g).
14

## Slide 15
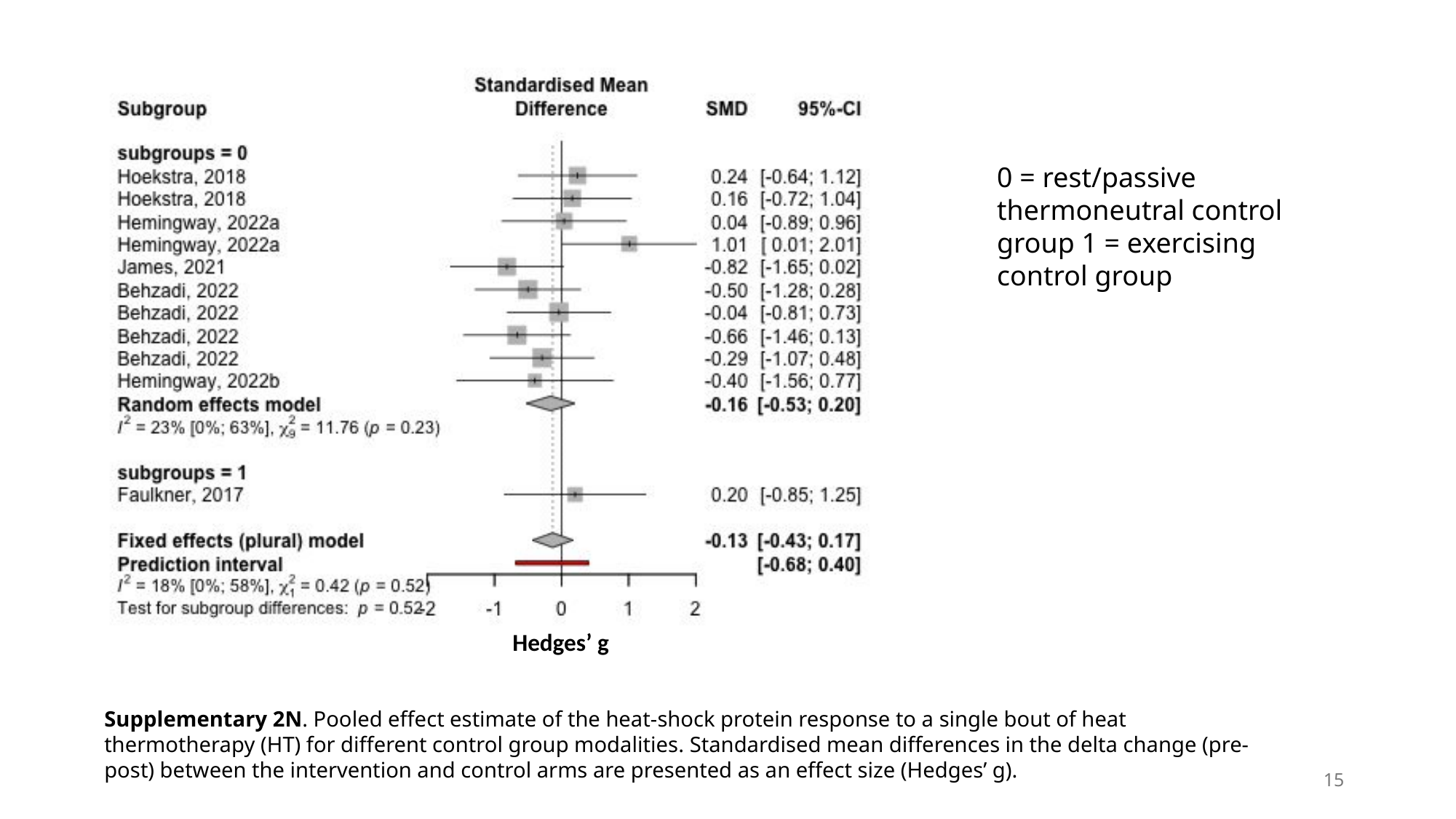

0 = rest/passive thermoneutral control group 1 = exercising control group
Hedges’ g
Supplementary 2N. Pooled effect estimate of the heat-shock protein response to a single bout of heat thermotherapy (HT) for different control group modalities. Standardised mean differences in the delta change (pre-post) between the intervention and control arms are presented as an effect size (Hedges’ g).
15

## Slide 16
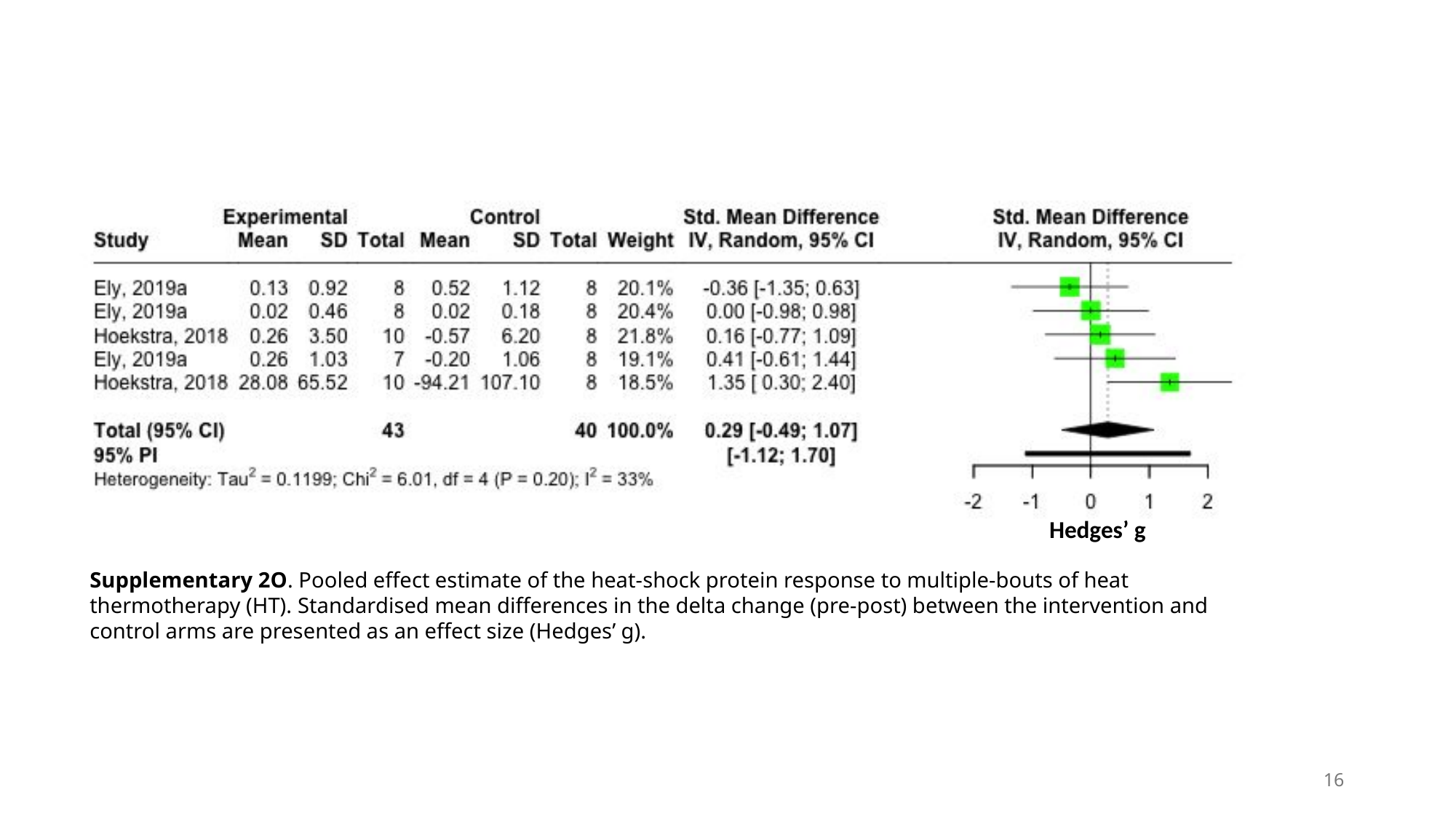

Hedges’ g
Supplementary 2O. Pooled effect estimate of the heat-shock protein response to multiple-bouts of heat thermotherapy (HT). Standardised mean differences in the delta change (pre-post) between the intervention and control arms are presented as an effect size (Hedges’ g).
16

## Slide 17
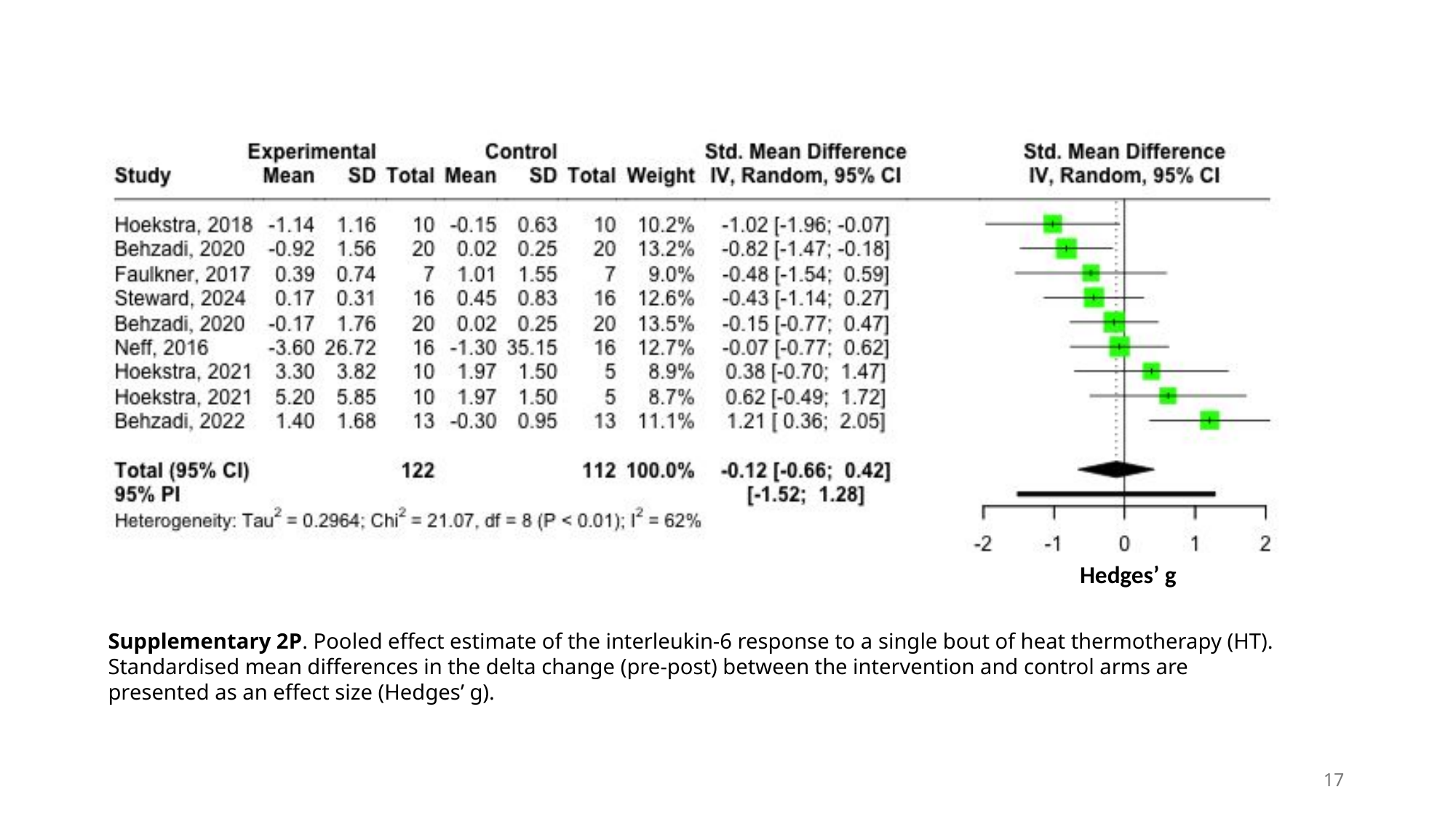

Hedges’ g
Supplementary 2P. Pooled effect estimate of the interleukin-6 response to a single bout of heat thermotherapy (HT). Standardised mean differences in the delta change (pre-post) between the intervention and control arms are presented as an effect size (Hedges’ g).
17

## Slide 18
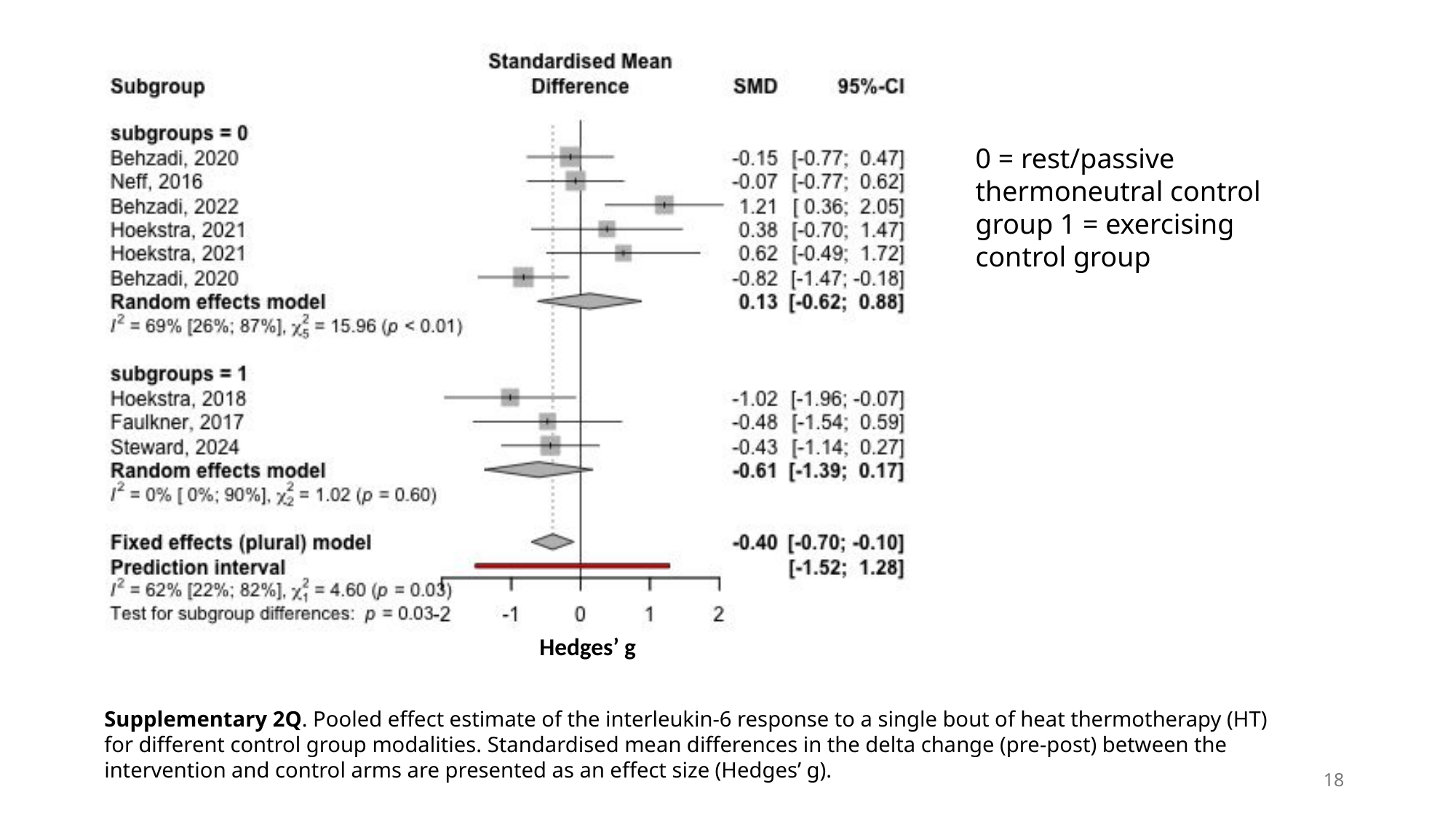

0 = rest/passive thermoneutral control group 1 = exercising control group
Hedges’ g
Supplementary 2Q. Pooled effect estimate of the interleukin-6 response to a single bout of heat thermotherapy (HT) for different control group modalities. Standardised mean differences in the delta change (pre-post) between the intervention and control arms are presented as an effect size (Hedges’ g).
18

## Slide 19
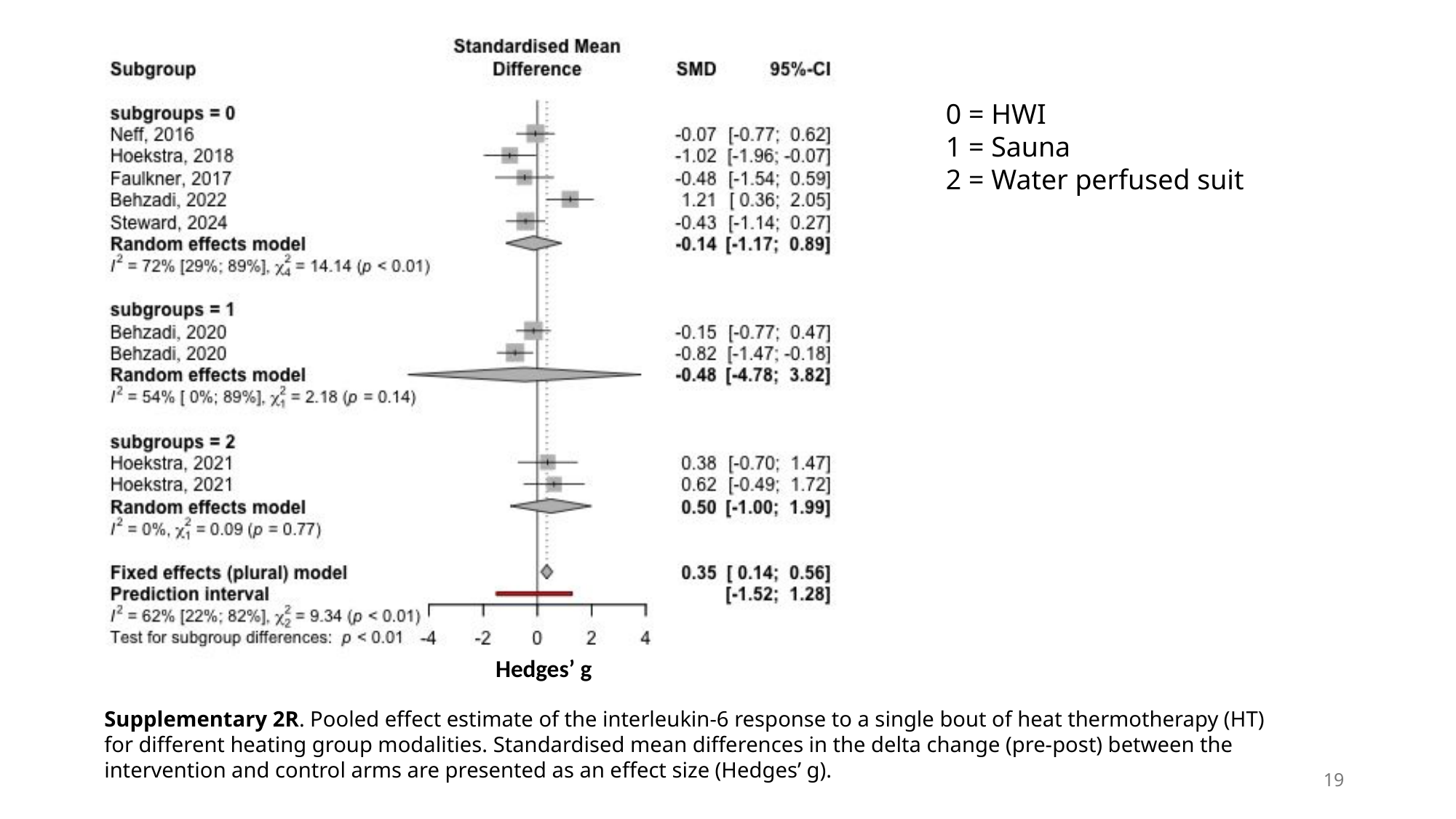

0 = HWI
1 = Sauna
2 = Water perfused suit
Hedges’ g
Supplementary 2R. Pooled effect estimate of the interleukin-6 response to a single bout of heat thermotherapy (HT) for different heating group modalities. Standardised mean differences in the delta change (pre-post) between the intervention and control arms are presented as an effect size (Hedges’ g).
19

## Slide 20
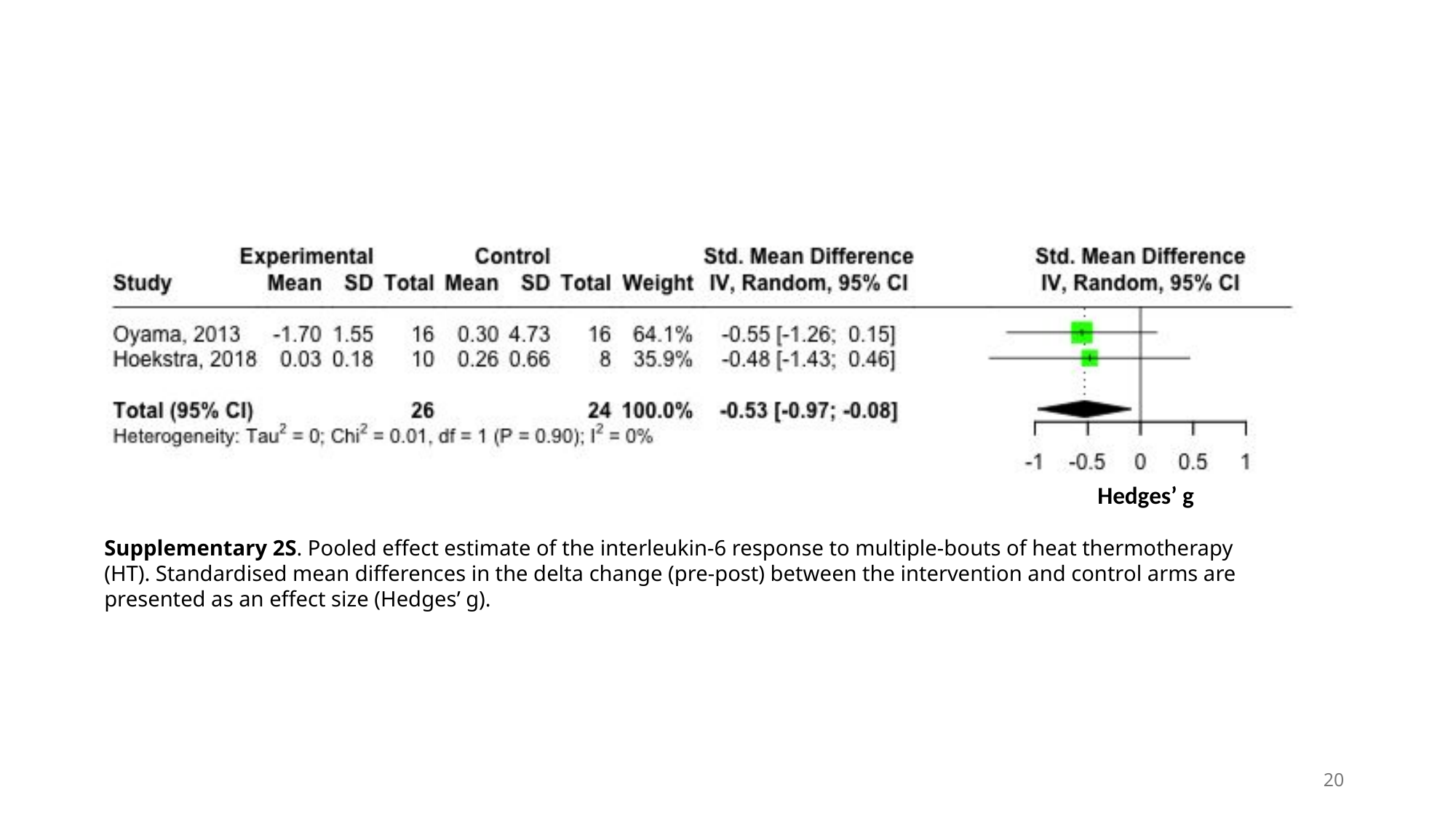

Hedges’ g
Supplementary 2S. Pooled effect estimate of the interleukin-6 response to multiple-bouts of heat thermotherapy (HT). Standardised mean differences in the delta change (pre-post) between the intervention and control arms are presented as an effect size (Hedges’ g).
20

## Slide 21
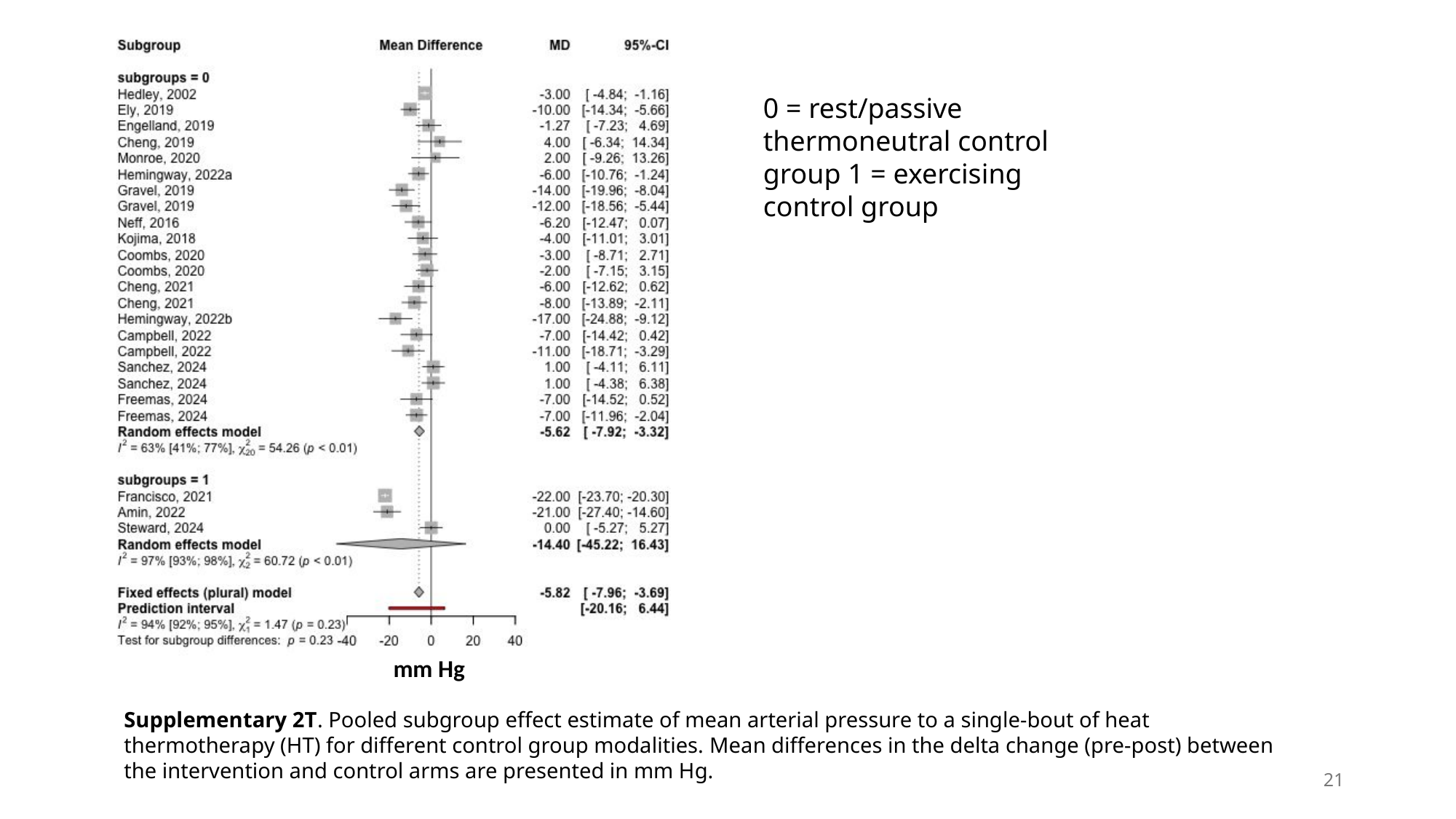

0 = rest/passive thermoneutral control group 1 = exercising control group
mm Hg
Supplementary 2T. Pooled subgroup effect estimate of mean arterial pressure to a single-bout of heat thermotherapy (HT) for different control group modalities. Mean differences in the delta change (pre-post) between the intervention and control arms are presented in mm Hg.
21

## Slide 22
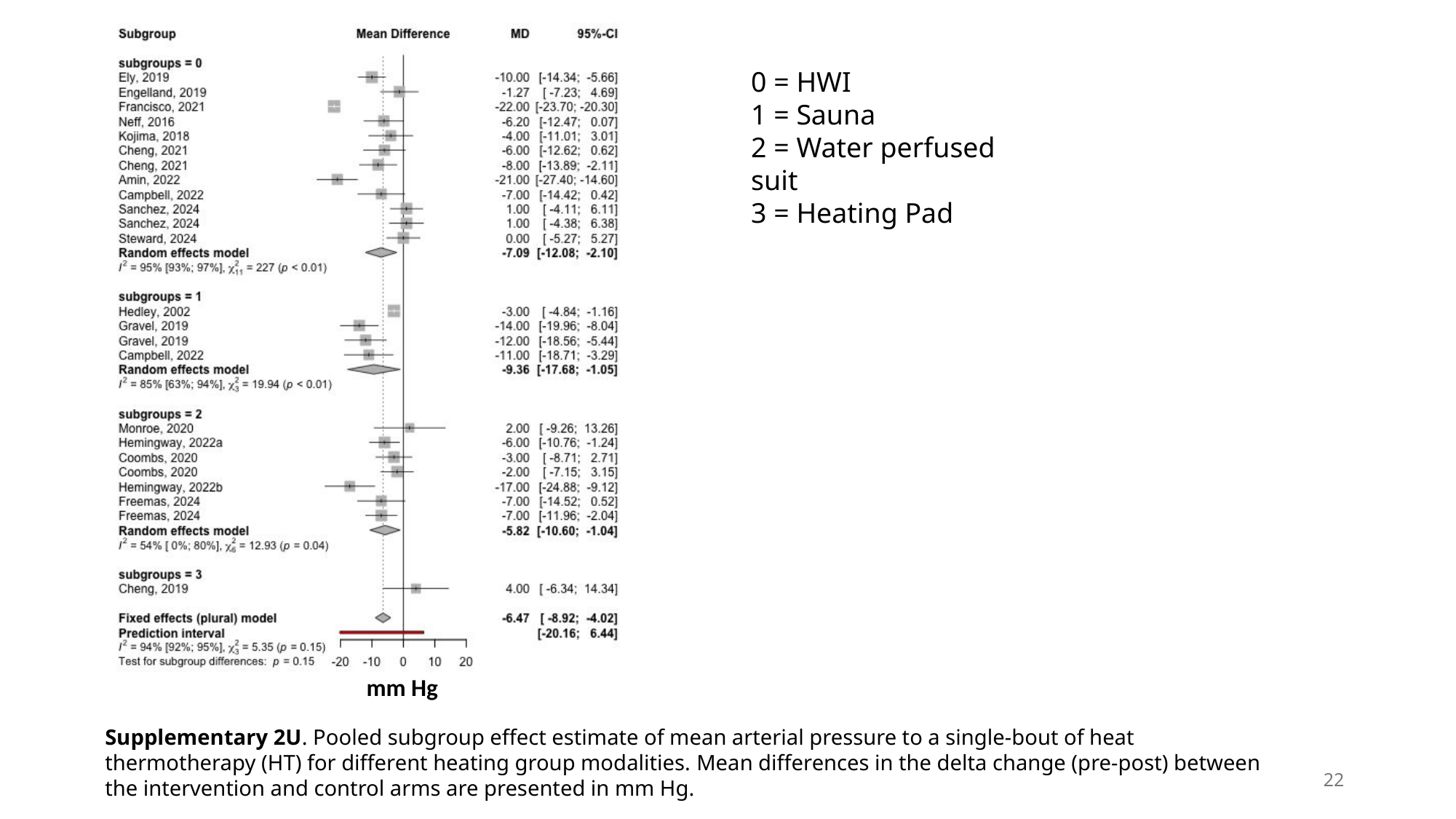

0 = HWI
1 = Sauna
2 = Water perfused suit
3 = Heating Pad
mm Hg
Supplementary 2U. Pooled subgroup effect estimate of mean arterial pressure to a single-bout of heat thermotherapy (HT) for different heating group modalities. Mean differences in the delta change (pre-post) between the intervention and control arms are presented in mm Hg.
22

## Slide 23
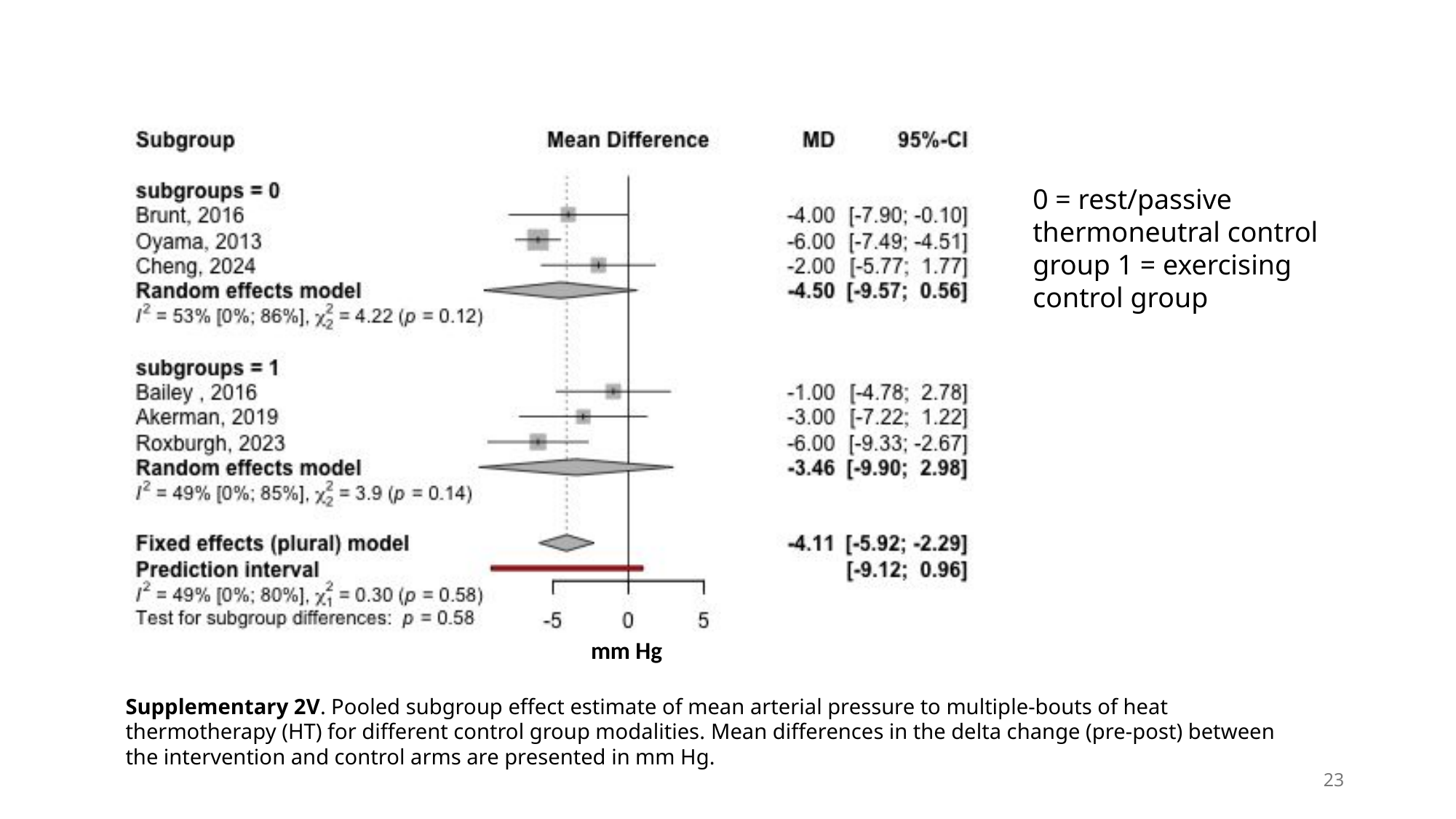

0 = rest/passive thermoneutral control group 1 = exercising control group
mm Hg
Supplementary 2V. Pooled subgroup effect estimate of mean arterial pressure to multiple-bouts of heat thermotherapy (HT) for different control group modalities. Mean differences in the delta change (pre-post) between the intervention and control arms are presented in mm Hg.
23

## Slide 24
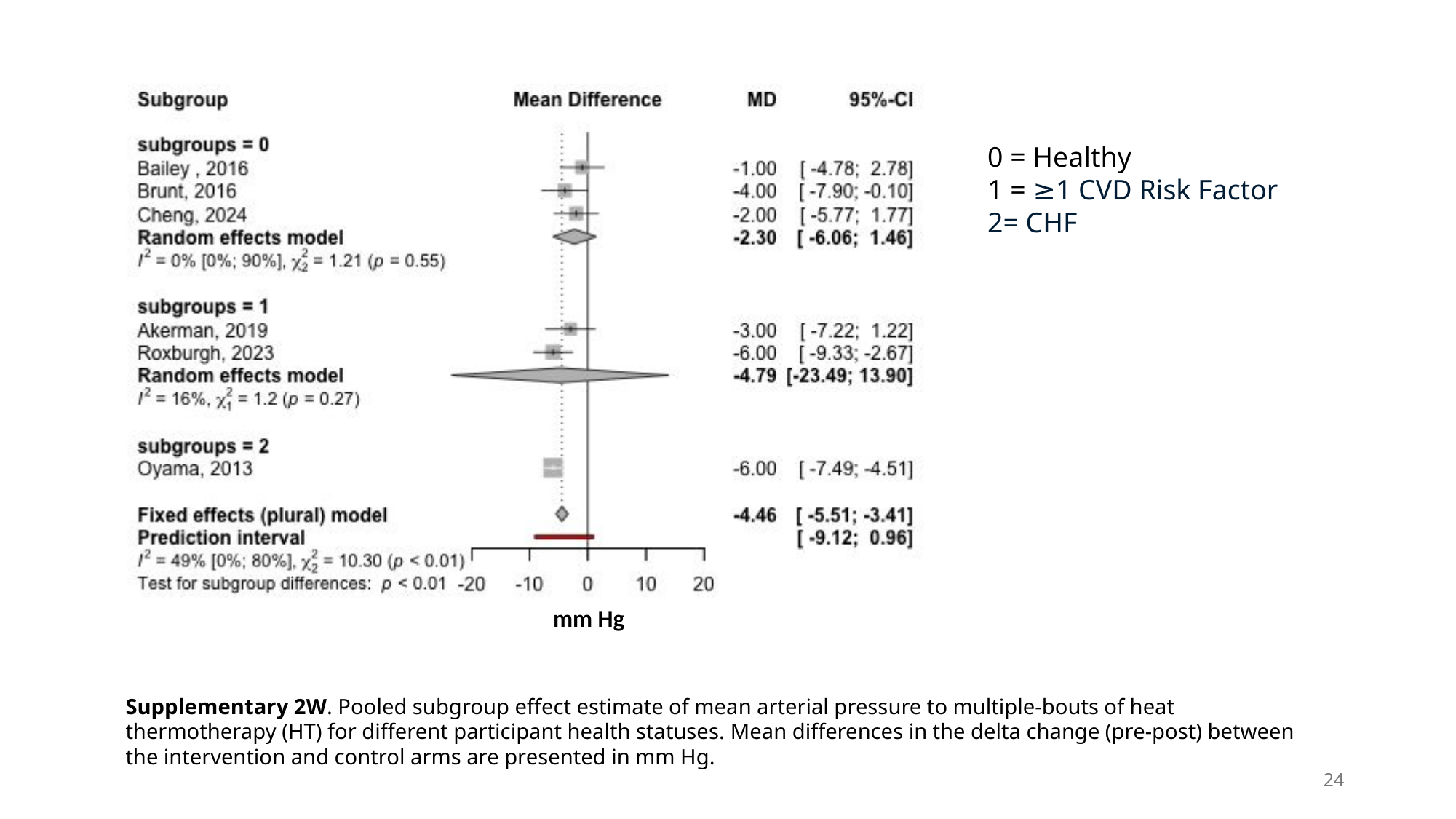

0 = Healthy
1 = ≥1 CVD Risk Factor
2= CHF
mm Hg
Supplementary 2W. Pooled subgroup effect estimate of mean arterial pressure to multiple-bouts of heat thermotherapy (HT) for different participant health statuses. Mean differences in the delta change (pre-post) between the intervention and control arms are presented in mm Hg.
24

## Slide 25
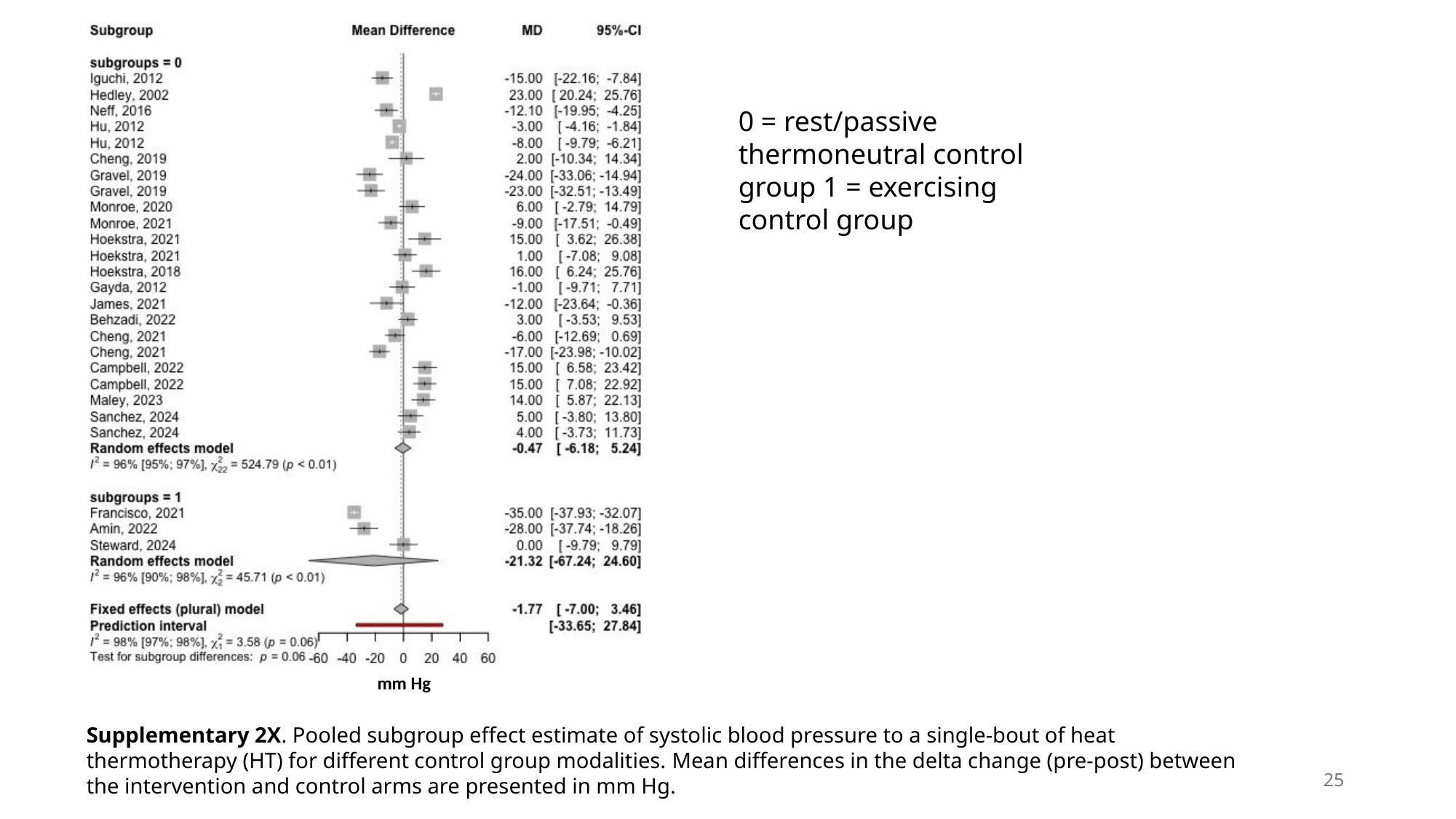

0 = rest/passive thermoneutral control group 1 = exercising control group
mm Hg
Supplementary 2X. Pooled subgroup effect estimate of systolic blood pressure to a single-bout of heat thermotherapy (HT) for different control group modalities. Mean differences in the delta change (pre-post) between the intervention and control arms are presented in mm Hg.
25

## Slide 26
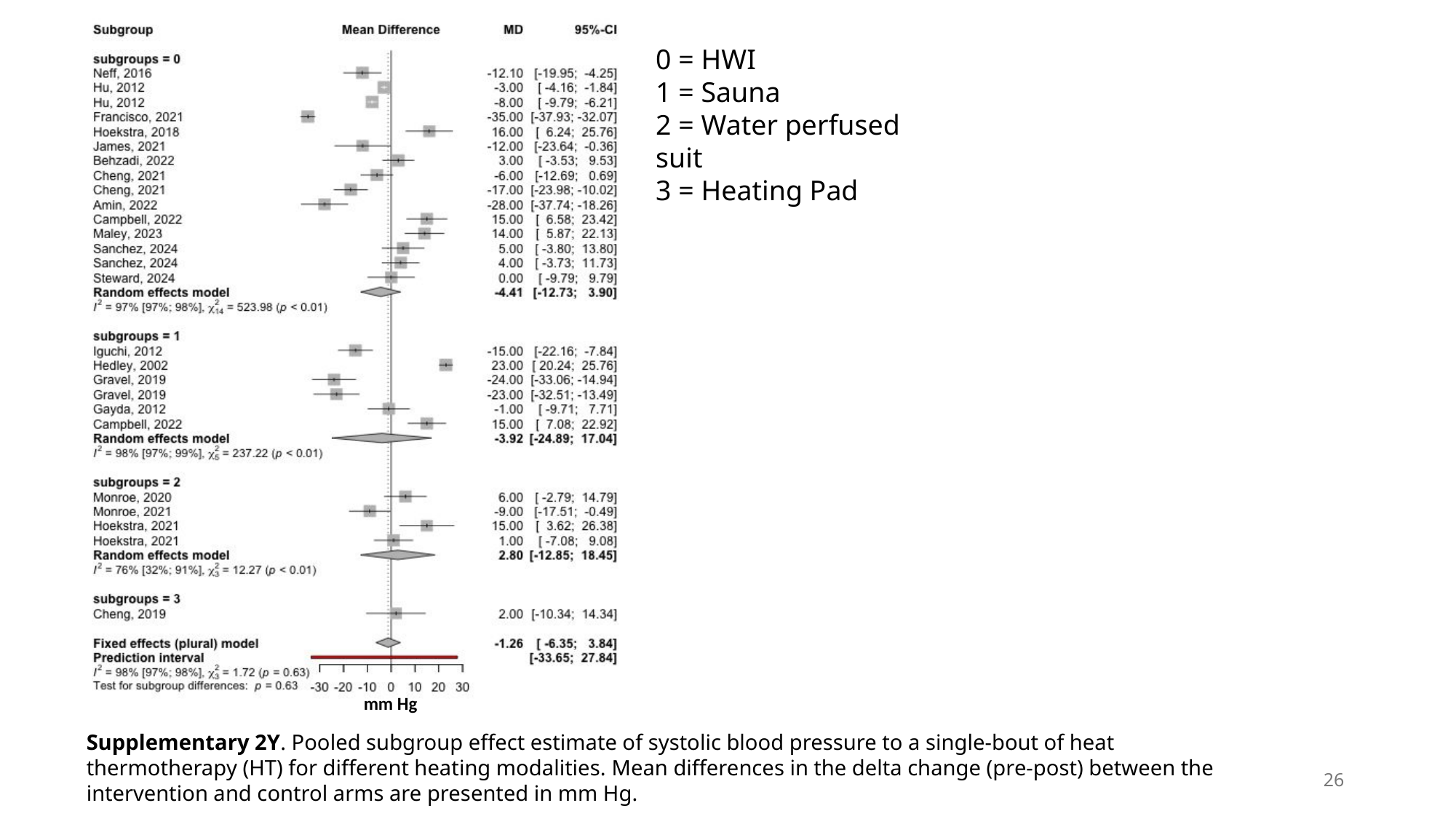

0 = HWI
1 = Sauna
2 = Water perfused suit
3 = Heating Pad
mm Hg
Supplementary 2Y. Pooled subgroup effect estimate of systolic blood pressure to a single-bout of heat thermotherapy (HT) for different heating modalities. Mean differences in the delta change (pre-post) between the intervention and control arms are presented in mm Hg.
26

## Slide 27
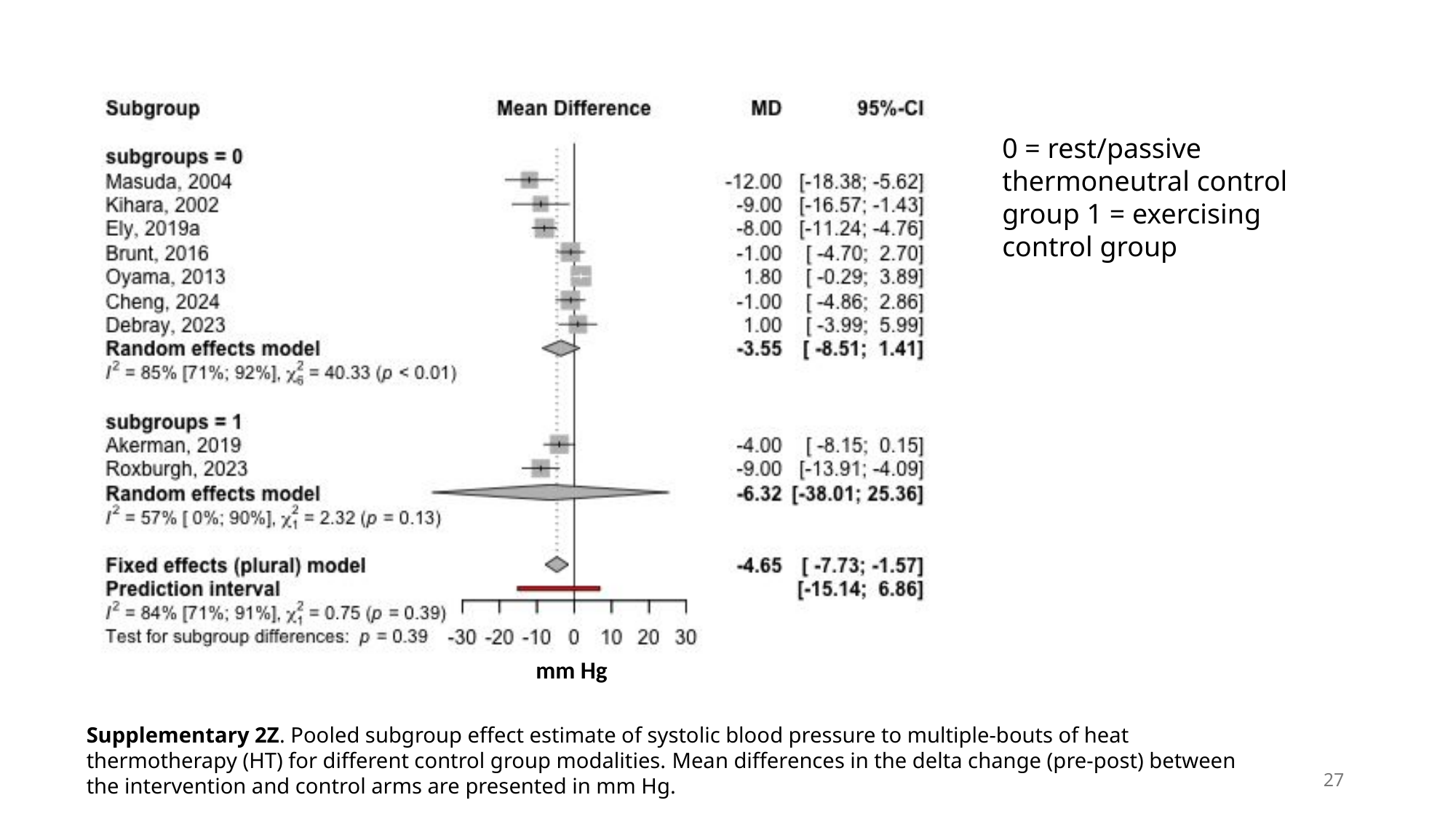

0 = rest/passive thermoneutral control group 1 = exercising control group
mm Hg
Supplementary 2Z. Pooled subgroup effect estimate of systolic blood pressure to multiple-bouts of heat thermotherapy (HT) for different control group modalities. Mean differences in the delta change (pre-post) between the intervention and control arms are presented in mm Hg.
27

## Slide 28
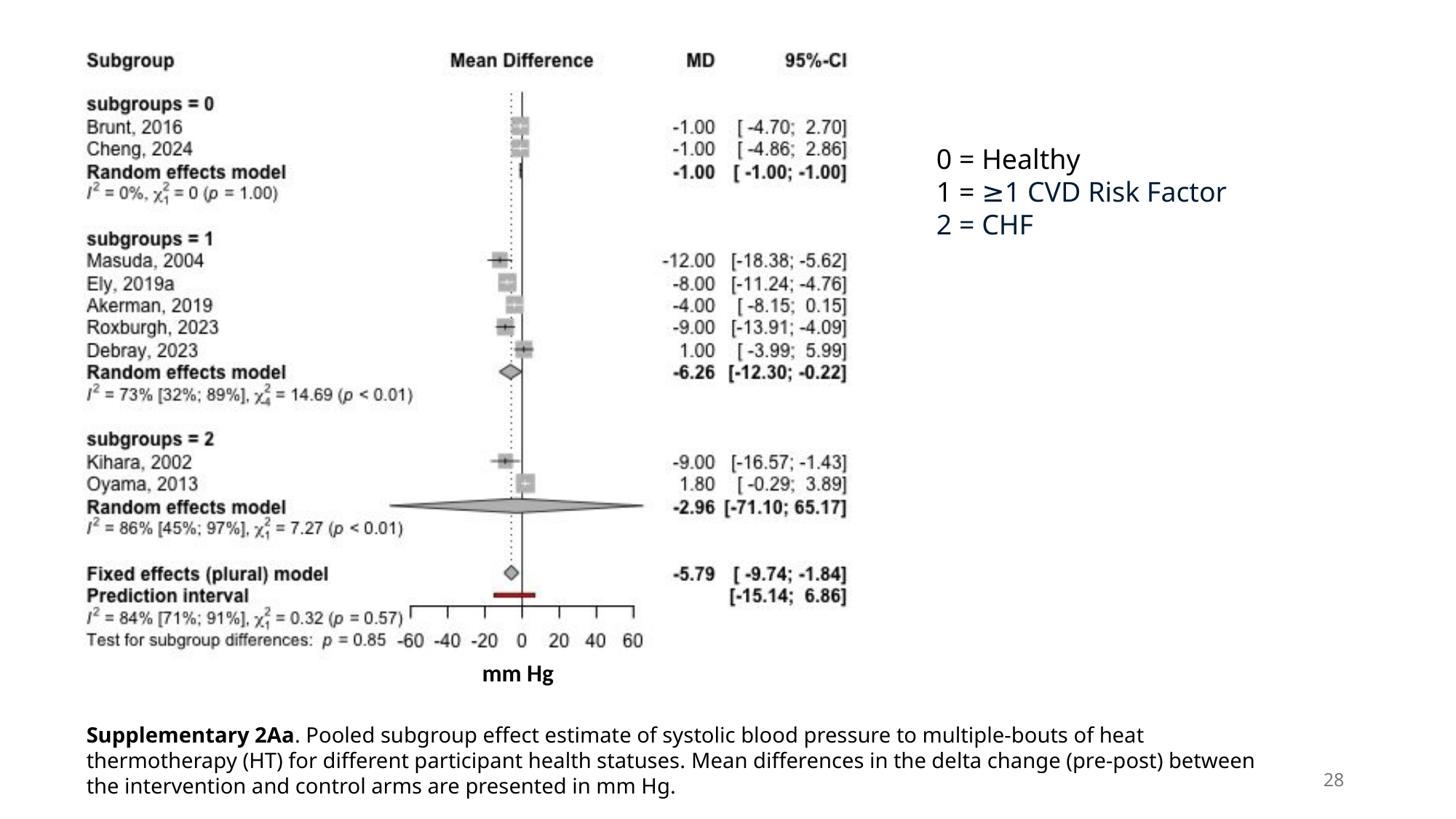

0 = Healthy
1 = ≥1 CVD Risk Factor
2 = CHF
mm Hg
Supplementary 2Aa. Pooled subgroup effect estimate of systolic blood pressure to multiple-bouts of heat thermotherapy (HT) for different participant health statuses. Mean differences in the delta change (pre-post) between the intervention and control arms are presented in mm Hg.
28

## Slide 29
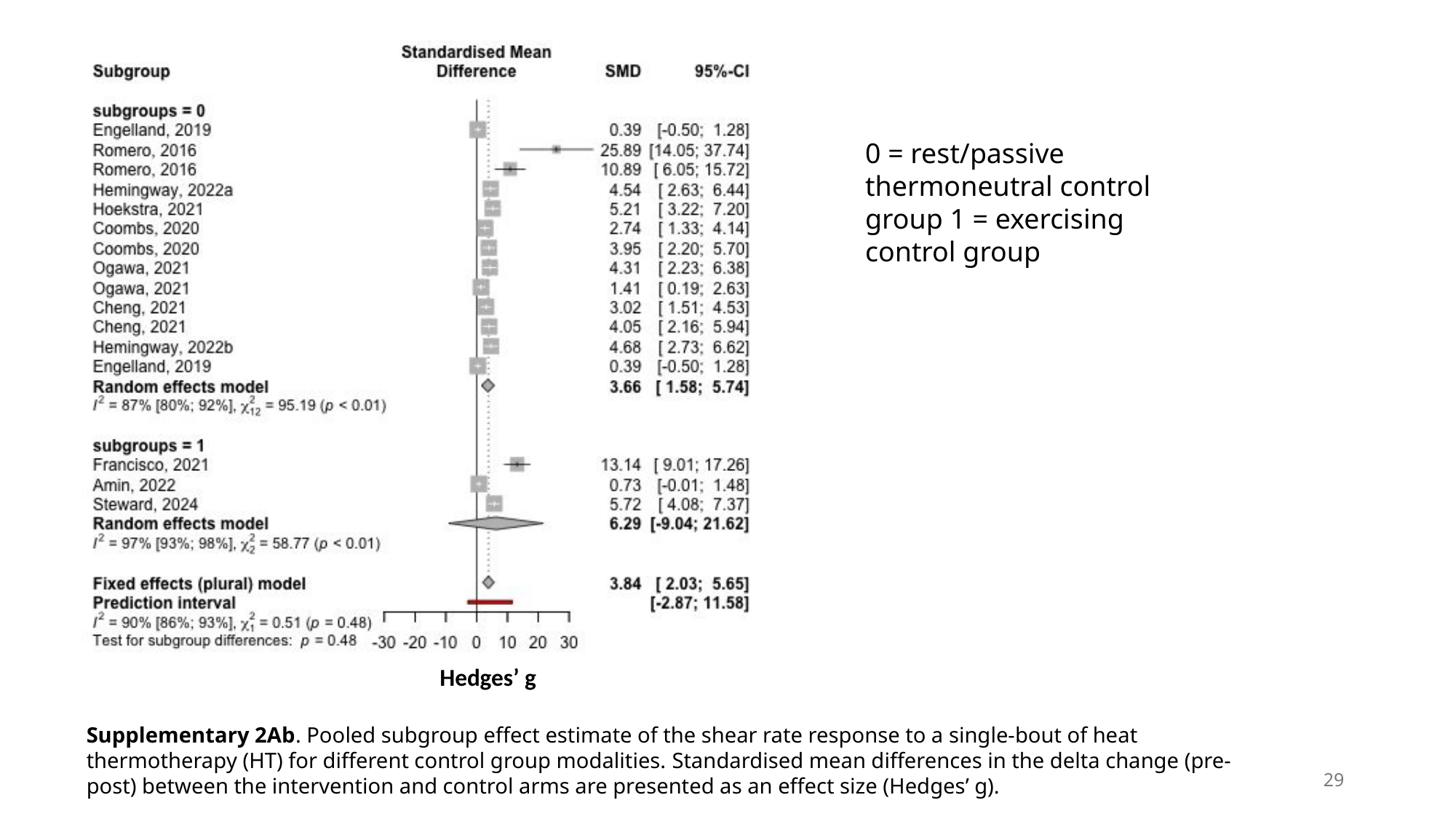

0 = rest/passive thermoneutral control group 1 = exercising control group
Hedges’ g
Supplementary 2Ab. Pooled subgroup effect estimate of the shear rate response to a single-bout of heat thermotherapy (HT) for different control group modalities. Standardised mean differences in the delta change (pre-post) between the intervention and control arms are presented as an effect size (Hedges’ g).
29
