# Supplementary material for: Heat thermotherapy to improve cardiovascular function and cardiometabolic health: A systematic review and meta‐analysis
Source: Exp Physiol. 2025 Oct 30:10.1113/EP092404. Online ahead of print. doi: 10.1113/EP092404 (PMC13394758; doi:10.1113/EP092404)
Supplement: Supplementary file 2 — Supporting Information [file EPH-9999-0-s001.docx]

**Supplementary Material**

**Supplement 1.** The inclusion criteria for the systematic review using the PICO Process.

| Population  Adult Humans | Intervention  Heat Thermotherapy | Comparison  Causational Study Design | Outcome  Cardiovascular & Cardiometabolic |
| --- | --- | --- | --- |
| Human  Adult  Hypertension  Overweight  Obese  Metabolic Syndrome  Metabolic Dysfunction  Insulin Resistant  Arteriosclerosis  Dyslipidaemia  Hyperglycaemia  Hyperinsulinemia  Cardiovascular Disease  Diabetes Mellitus Type Two  Sedentary Lifestyle | Induced Hyperthermia  Balneology  Sauna  Hydrotherapy  Hot Temperature Immersion  Hot Springs  Hot Water Immersion Heating  Passive Heating Infrared Sauna Sweating  Thermal Exposure Heat Stress  Finnish Sauna  Hot Bath ThermORegulation Heat Acclimation | Randomised Control Trial  Control Trial  Crossover Studies  Not Observational Studies  Journals Only  English Only | Flow Mediated Dilation  Microvascular Function  Arterial Stiffness  Blood Pressure  Ischemic Conditioning  Endothelial Function  IL-6  C-reactive protein  Heat Shock Protein  Glucose |

*Note.* PICO (Participant, Intervention, Comparison and Outcome) framework. From: Schardt C, Adams MB, Owens T, Keitz S, Utilization of the PICO framework to improve searching PubMed for clinical questions. BMC Medical Informatics and Decision Making 7(1): 16. doi:10.1186/1472-6947-7-16.

**Supplement 2**. Search terms for the MEDLINE, EMBASE and Web of Science based from the PICO.

| **Section** | **MEDLINE** | **EMBASE** | **Web of Science** |
| --- | --- | --- | --- |
| **Population** | Humans/ **OR** Adult/ OR Aged/ OR Middle Aged/ OR Young Adult/ **OR** Hypertension/ **OR** Overweight/ **OR** Obesity/ **OR** Cardiovascular Diseases/ **OR** Insulin Resistance/ **OR** Metabolic Syndrome/ **OR** Diabetes Mellitus, Type 2/ **OR** Sedentary Lifestyle/ **OR** Arteriosclerosis/ **OR** Metabolic Dysfunction.Mp. **OR** Hyperglycemia/ **OR** Hyperinsulinism/ **OR** Dyslipidemias/ | Human/ **OR** Adult/ **OR** Hypertension/ **OR** Obesity/ **OR** Cardiovascular Disease/ **OR** Metabolic Syndrome X/ **OR** Insulin Resistance/ **OR** Diabetes Mellitus/ **OR** Inflammation/ **OR** Sedentary Life Style/ **OR** Atherosclerosis/ **OR** Young Adult/ **OR** Aged/ **OR** Metabolic Dysfunction.Mp. **OR** Hyperglycemia/ **OR**  Hyperinsulinemia/ **OR** Dyslipidemia/ | ALL = (Humans **OR** Adult **OR** Aged **OR** Hypertension **OR** Overweight **OR** Obes* **OR** Cardiovascular Disease* **OR** Insulin Resistance **OR** Metabolic* **OR** Type 2 Diabetes Mellitus **OR** Sedentary Lifestyle **OR** Arteriosclerosis **OR** Hyperglycemia |
| **Intervention** | Hyperthermia, Induced/ **OR** Balneology/ **OR** Ammotherapy/ **OR** Baths/ **OR** Steam Bath/ **OR** Sauna.Mp. **OR** Hydrotherapy/ **OR** Ammotherapy/ **OR** Diathermy/ **OR** Steam Bath/ **OR** *Immersion/ **OR** Hot Springs/ **OR** Hot Water Immersion.Mp. **OR** Exp Heating/ **OR** Passive Heat*.Mp. **OR** Infrared Sauna.Mp. **OR** Sweating/ **OR** Hot Temperature/ **OR** Heat Stress.Mp. **OR** Heat-Shock Response/ **OR** Finnish Sauna.Mp. **OR** Thermoregulation/ **OR** Heat Acclimation **OR** Heating/Mt **OR** *Hyperthermia, Induced/Mt | Balneotherapy/ **OR** Hydrotherapy/ **OR** Sauna/ **OR** Spa Treatment/ **OR** Heat Acclimatization/ **OR** *Heat/ **OR** Temperature Related Phenomena/ **OR** *Thermotherapy/ **OR** Diathermy/ **OR** Microwave Thermotherapy/ **OR** Heat Stress/ **OR** Sweating/ **OR** Passive Heat*.Mp. **OR** *Bath.Mp. **OR** Heating/ **OR** Heat Acclimation.Mp. **OR** Heat Shock Response/ **OR** Thermoregulation/ **OR** Heat Acclimation.Mp. | ALL = (Bath* **OR** Sauna **OR** Hydrotherapy **OR** Hot Springs **OR** Hot Water Immersion **OR** Heating **OR** Passive Heating **OR** Infrared Sauna **OR** Thermal Exposure **OR** Heat Stress **OR** Finnish Sauna **OR** Thermoregulation **OR** Heat Acclimation **OR** Heat Therapy **OR** Sweating |
| **Comparison** | Cross-Over.Mp. **OR** Random Allocation/ **OR** Randomized Controlled Trials as Topic/ **OR** Controlled Clinical Trials as Topic/ | Randomized Controlled Trial/ **OR** Crossover.Mp. **OR** Randomization.Mp. | All = (Crossover **OR** Randomi*Ed **OR** Randomi*ed Controlled Trial **OR** Controlled Clinical Trial **OR** Parallel **OR** Random Allocation) |
| **Outcome** | **Cardiovascular**  Microcirculation **OR** Cerebrovascular Circulation/ **OR** Diagnostic Techniques, Cardiovascular/ **OR** Microscopic Angioscopy/ **OR** Ultrasonography/ **OR** Echocardiography/ **OR** Ultrasonography, Doppler/ **OR** Echocardiography, Doppler/ **OR** Ultrasonography, Doppler, Duplex/ **OR** Ultrasonography, Doppler, Pulsed/ **OR** Flow Mediated Dilation.Mp **OR** Endothelial Progenitor Cells/ **OR** Shear Stress.Mp. **OR** Vascular Stiffness/ **OR** Microscopic Angioscopy/ **OR** Blood Pressure/ **OR** Ischemic Preconditioning/ **OR** Endothelium Vascular/ **OR** Reperfusion/ **OR** Endothelial Function.Mp. **OR** Cutaneous Vascular Function.Mp. **OR** Vascular Function.Mp. **OR** Cutaneous Microvascular Function.Mp. **OR** Nitric Oxide/ **OR** Laser Doppler Flowmetry/ **OR** Endothelial Dysfunction.Mp. | **Cardiovascular**  Microcirculation/ **OR** Brain Circulation/ OR Brain Microcirculation/ **OR** Doppler Flowmetry/ **OR** Cardiovascular System Examination/ **OR** Cardiovascular Function/ **OR** Endothelial Progenitor Cell/ **OR** Shear Stress/ **OR** Arterial Stiffness/ **OR** Glycosylated Hemoglobin/ **OR** Angioscopy/ **OR** Blood Pressure/ **OR** Ischemic Preconditioning/ **OR** Reperfusion/ **OR** Laser Doppler Flowmetry/ **OR** Endothelium Vascular/ **OR** Endothelial Dysfunction/ OR Endothelial Function.Mp. **OR** Arterial Wall Thickness/ **OR** Cutaneous Vascular Function.Mp. **OR** Vascular Function.Mp. **OR** Cutaneous Microvascular Function.Mp. **OR** Nitric Oxide/ **OR** Endothelial Dysfunction | **Cardiovascular**  ALL = (Microcirculation **OR** Cerebrovascular Circulation **OR** Ultrasound **OR** Shear Rate **OR** Arterial Stiffness **OR** Blood Pressure **OR** Endothelial Function **OR** Vascular Function **OR** Endothelial Dysfunction **OR** Ischemic Preconditioning **OR** Arterial Wall Thickness **OR** Cutaneous Vascular Function |
|  | **Cardiometabolic**  Insulin/ **Or** Receptor, Insulin **Or** Receptors, Vascular Endothelial **Or** Blood Glucose/ **Or** Glycated Hemoglobin A/ **Or** C-Reactive Protein/ **Or** Tumor Necrosis Factor-Alpha/ **Or** Interleukin-6/ **Or** Interleukin-10 **Or** Heat-Shock Proteins/ **Or** Chaperonins/ **Or** Heat-Shock Proteins, Small/ **Or** Hsp40 Heat-Shock Proteins/ **Or** Hsp47 Heat-Shock Proteins/ **Or** Hsp70 Heat-Shock Proteins/ **Or** Hsp90 Heat-Shock Proteins/ **Or** Immunity/ **Or** Blood Glucose.Mp. **Or** Oxidative Stress/ **Or** Postprandial Period/ **Or** Blood Lipids.Mp. **Or** Glycation End Products, Advanced/ | **Cardiometabolic**  Human Insulin **Or** Insulin Receptor/ **Or** Glucose Blood Level/ Or Glucose Level/ **Or** Glycosylated Hemoglobin/ **Or** Hemoglobin A1c/ **Or** C Reactive Protein/ **Or** Insulin Sensitivity/ **Or** Interleukin 6/ **Or** Tumor Necrosis Factor/ **Or** Interleukin 10/ **Or** Heat Shock Protein/ **Or** Chaperonin 60/ **Or** Heat Shock Cognate Protein 70/ **Or** Heat Shock Protein 40/ **Or** Heat Shock Protein 47/ **Or** Heat Shock Protein 70/ **Or** Heat Shock Protein 72/ **Or** Heat Shock Protein 90/ **Or** Heat Shock Protein 90 Alpha/ **Or** Small Heat Shock Protein/ **Or** Blood Glucose Level/ **Or** Oxidative Stress/ **Or** Lipid Blood Level/ **Or** Postprandial State/ **Or** Advanced Glycation End Products/ | **Cardiometabolic**  ALL = (Blood Glucose **OR** Insulin **OR** Insulin Receptor **OR** C-Reactive Protein **OR** Interleukin 6 **OR** Heat Shock Protein* **OR** Oxidative Stress **OR** Postprandial Glucose Oxidative Stress **OR** Nitric Oxide) |
| **Limit** | Limit To English | Limit To English Language | None |

*Note.* Each section, such as population, was entered into the database. Then, each section was combined (Population AND Intervention AND Comparison AND Intervention) before entering the limit (if applicable) to complete the database search. Once completed, papers were uploaded to EndNote, and the screening processes took place.

**Supplement 3**. Risk of bias assessment for publications included in the Meta-Analysis

| **Reference** | **Bias arising from the randomisation process** | **Bias due to deviations from intended interventions** | **Bias due to missing outcome data** | **Bias in the measurement of the outcome** | **Bias in the selection in the repORted result** | **Overall Risk of Bias Grade** |
| --- | --- | --- | --- | --- | --- | --- |
| Akerman et al., 2019 | Some Concerns | Low Risk | Low Risk | Low Risk | Some Concerns | Some Concerns |
| Amin et al., 2021 | High Risk | Low Risk | Low Risk | Low Risk | Some Concerns | High Risk |
| Bailey et al., 2016 | Low Risk | Low Risk | Low Risk | Low Risk | Some Concerns | Some Concerns |
| Behazdi et al., 2020 | High Risk | Low Risk | Low Risk | Low Risk | Some Concerns | High Risk |
| Behazdi et al., 2022 | Some Concerns | Low Risk | Low Risk | Low Risk | Some Concerns | Some Concerns |
| Brunt et al., 2016a | High Risk | Low Risk | Low Risk | Low Risk | Some Concerns | High Risk |
| Brunt et al., 2016b | Some Concerns | Low Risk | Low Risk | Low Risk | Some Concerns | Some Concerns |
| Campbell et al., 2022 | Some Concerns | Low Risk | Low Risk | Low Risk | Some Concerns | Some Concerns |
| Cheng et al., 2019 | Some Concerns | Low Risk | Low Risk | Low Risk | Some Concerns | Some Concerns |
| Cheng et al., 2021 | Some Concerns | Low Risk | Low Risk | Low Risk | Some Concerns | Some Concerns |
| Cheng et al., 2024 | Low Risk | Low Risk | Low Risk | Low Risk | Low Risk | Low Risk |
| Coombs et al., 2021 | Some Concerns | Low Risk | Low Risk | Low Risk | Some Concerns | Some Concerns |
| Debray et al., 2023 | Low Risk | Low Risk | Low Risk | Low Risk | Low Risk | Low Risk |
| Ely et al., 2019a | High Risk | Low Risk | Low Risk | Low Risk | Some Concerns | High Risk |
| Ely et al., 2019b | Some Concerns | Low Risk | Low Risk | Low Risk | Some Concerns | Some Concerns |
| Engelland et al., 2019 | Some Concerns | Low Risk | Low Risk | Low Risk | Some Concerns | Some Concerns |
| Fatahi et al., 2023 | Some Concerns | Low Risk | Low Risk | Low Risk | Low Risk | Low Risk |
| Faulkner et al., 2017 | Some Concerns | Low Risk | Low Risk | Low Risk | Some Concerns | Some Concerns |
| Francisco et al., 2021 | Some Concerns | Low Risk | Low Risk | Low Risk | Some Concerns | Some Concerns |
| Freemas et al., 2024 | Some Concerns | Low Risk | Low Risk | Low Risk | Some Concerns | Some Concerns |
| Gayda et al., 2012 | Some Concerns | Low Risk | Low Risk | Low Risk | Some Concerns | Some Concerns |
| Gravel et al., 2019 | Some Concerns | Low Risk | Low Risk | Low Risk | Some Concerns | Some Concerns |
| Hedley et al., 2002 | Some Concerns | Low Risk | Low Risk | Low Risk | Some Concerns | Some Concerns |
| Hemingway 2022a | Some Concerns | Low Risk | Low Risk | Low Risk | Some Concerns | Some Concerns |
| Hemingway 2022b | High Risk | Low Risk | Low Risk | Low Risk | Some Concerns | High Risk |
| Hoekstra et al., 2018 | Some Concerns | Low Risk | Low Risk | Low Risk | Some Concerns | Some Concerns |
| Hoekstra et al., 2021 | Some Concerns | Low Risk | Low Risk | Low Risk | Some Concerns | Some Concerns |
| Hu et al., 2012 | Some Concerns | Low Risk | Some Concerns | Low Risk | Some Concerns | Some Concerns |
| Iguchi et al., 2012 | Some Concerns | Low Risk | Low Risk | Low Risk | Some Concerns | Some Concerns |
| Imamura et al., 2001 | High Risk | Low Risk | Low Risk | Low Risk | Some Concerns | High Risk |
| James et al., 2021 | Some Concerns | Low Risk | Low Risk | Low Risk | Some Concerns | Some Concerns |
| Kihara et al., 2002 | Some Concerns | Low Risk | Low Risk | Low Risk | Some Concerns | Some Concerns |
| Kimball et al., 2018 | Some Concerns | Low Risk | Low Risk | Low Risk | Some Concerns | Some Concerns |
| Kojima et al., 2018 | Some Concerns | Low Risk | Low Risk | Low Risk | Some Concerns | Some Concerns |
| Leicht et al., 2019 | Some Concerns | Low Risk | Low Risk | Low Risk | Some Concerns | Some Concerns |
| Maley et al., 2023 | Some Concerns | Low Risk | Low Risk | Low Risk | Some Concerns | Some Concerns |
| Masuda et al., 2004 | Low Risk | Low Risk | Low Risk | Low Risk | Some Concerns | Some Concerns |
| McGarity-Shipley et al., 2021 | Some Concerns | Low Risk | Low Risk | Low Risk | Some Concerns | Some Concerns |
| Monroe et al., 2020 | Some Concerns | Low Risk | Low Risk | Low Risk | Some Concerns | Some Concerns |
| Monroe et al., 2021 | Some Concerns | Low Risk | Low Risk | Low Risk | Some Concerns | Some Concerns |
| Neff et al., 2016 | Some Concerns | Low Risk | Low Risk | Low Risk | Some Concerns | Some Concerns |
| Ogawa et al., 2021 | Some Concerns | Low Risk | Low Risk | Low Risk | Some Concerns | Some Concerns |
| Olah et al., 2011 | Some Concerns | Low Risk | Low Risk | Low Risk | Some Concerns | Some Concerns |
| Oyama et al., 2013 | Some Concerns | Low Risk | Low Risk | Low Risk | Some Concerns | Some Concerns |
| Qiu et al., 2014 | Some Concerns | Low Risk | Low Risk | Low Risk | Some Concerns | Some Concerns |
| Romero et al., 2017 | Some Concerns | Low Risk | Low Risk | Low Risk | Some Concerns | Some Concerns |
| Roxburgh et al., 2023 | Low Risk | Low Risk | Low Risk | Low Risk | Low Risk | Low Risk |
| Sanchez et al., 2024 | Some Concerns | Low Risk | Low Risk | Low Risk | Some Concerns | Some Concerns |
| Schenaarts et al., 2024 | Low Risk | Low Risk | Low Risk | Low Risk | Some Concerns | Some Concerns |
| Steward et al., 2024 | Some Concerns | Low Risk | Low Risk | Low Risk | Some Concerns | Some Concerns |
| Texeira et al., 2017 | Some Concerns | Low Risk | Low Risk | Low Risk | Some Concerns | Some Concerns |

*Note.* To be considered a study with an overall low risk profile, all sections must be assessed as low risk. To be considered a study with an overall risk profile of some concerns, all sections must be either low risk or have some concerns. Any domain regarded as high risk will result in the entire study being classified as high risk overall.

**Supplement 4.** Summary of the risk of bias scores.

| **Risk of Bias Domain** | **High Risk Papers** | **Some Concerns** | **Low Risk** |
| --- | --- | --- | --- |
| **Risk of bias arising from the randomisation process** | **n = 6** | **n = 40** | **n = 5** |
| Risk of bias due to deviations from the intended interventions | **n = 0** | **n = 0** | **n = 51** |
| Missing outcome data | **n = 0** | **n = 1** | **n = 50** |
| Risk of bias in measurement of the outcome | **n = 0** | **n = 0** | **n = 51** |
| Risk of bias in selection of the reported result | **n = 0** | **n = 5** | **n = 46** |
| **Overall Risk of Bias** | **n = 6** | **n = 41** | **n = 4** |
